# Involvement of Fgf2-mediated tau protein phosphorylation in cognitive deficits induced by sevoflurane in aged rats

**DOI:** 10.1186/s10020-024-00784-0

**Published:** 2024-03-16

**Authors:** Xin Xie, Xiaomin Zhang, Songze Li, Wei Du

**Affiliations:** Department of Anesthesiology, Cancer Hospital of China Medical University, Liaoning Cancer Hospital & Institute, No. 44, Xiaoheyan Road, Dandong District, Liaoning Province, Shenyang 110042 P. R. China

**Keywords:** Aged rats, Sevoflurane, Cognitive impairment, Transcriptomic sequencing, Fgf2, Gsk3b, Tau protein phosphorylation

## Abstract

**Objective:**

Anesthetics have been linked to cognitive alterations, particularly in the elderly. The current research delineates how Fibroblast Growth Factor 2 (Fgf2) modulates tau protein phosphorylation, contributing to cognitive impairments in aged rats upon sevoflurane administration.

**Methods:**

Rats aged 3, 12, and 18 months were subjected to a 2.5% sevoflurane exposure to form a neurotoxicity model. Cognitive performance was gauged, and the GEO database was employed to identify differentially expressed genes (DEGs) in the 18-month-old cohort post sevoflurane exposure. Bioinformatics tools, inclusive of STRING and GeneCards, facilitated detailed analysis. Experimental validations, both in vivo and in vitro, examined Fgf2’s effect on tau phosphorylation.

**Results:**

Sevoflurane notably altered cognitive behavior in older rats. Out of 128 DEGs discerned, Fgf2 stood out as instrumental in regulating tau protein phosphorylation. Sevoflurane exposure spiked Fgf2 expression in cortical neurons, intensifying tau phosphorylation via the PI3K/AKT/Gsk3b trajectory. Diminishing Fgf2 expression correspondingly curtailed tau phosphorylation, neurofibrillary tangles, and enhanced cognitive capacities in aged rats.

**Conclusion:**

Sevoflurane elicits a surge in Fgf2 expression in aging rats, directing tau protein phosphorylation through the PI3K/AKT/Gsk3b route, instigating cognitive aberrations.

**Supplementary Information:**

The online version contains supplementary material available at 10.1186/s10020-024-00784-0.

## Introduction

Age-related cognitive impairment stands as a neurodegenerative condition tightly linked with the aging process, resulting in the deterioration of cognitive faculties (Cooley et al. 2023). This ailment not only disrupts the daily life of affected individuals but also exacts a substantial toll on their families and society at large (Krüger 2018). Emerging research has shed light on the potential contribution of specific anesthetic agents, notably sevoflurane, to the development of postoperative cognitive dysfunction (Narayan et al. 2021). Managing cognitive impairment in elderly patients poses formidable challenges within clinical realms (Clemency et al. 2022). The etiology of this ailment is intricate, often intertwining with other medical conditions, introducing intricacies into the selection of suitable treatment modalities (Amirghasemi et al. 2021). Furthermore, the effectiveness of various therapeutic medications remains uncertain, further complicating the therapeutic landscape (Müller et al. 2021). In terms of prognosis, a significant number of elderly individuals afflicted by cognitive impairment experience a progressive decline, significantly diminishing their overall quality of life (McCrea et al. 2021; Wang et al. 2021a; Van Meter et al. 2021). This also underscores the augmented demand for family and medical support to fulfill their fundamental daily requirements (Harmer et al. 2023). The administration of anesthesia drugs such as sevoflurane is associated with an elevated susceptibility to postoperative neurocognitive disorders, necessitating enhanced prudence on the part of anesthesiologists when devising anesthetic strategies for elderly patients (Saranteas et al. 2019).

High-throughput transcriptomic sequencing represents a potent investigative tool in the study of neurological disorders, enabling the comprehensive detection of gene expression modifications associated with these conditions (Rogawski et al. 2017). This advanced technology empowers researchers to precisely pinpoint and quantitatively assess the genes and signaling pathways that hold significance in disease progression (Efferth and Oesch 2021). The integration of high-throughput transcriptomics sequencing holds substantial promise in uncovering the enigmatic molecular mechanisms underpinning anesthesia-induced cognitive impairment in older adults, an area characterized by its lack of clarity (Pant et al. 2021). This innovative methodology aids in identifying gene variations linked to anesthesia, thereby providing insights into a deeper understanding of how anesthetics impact the function and structure of the aging brain (Severe Covid-19 GWAS Group et al. 2020). This research not only introduces innovative approaches for early disease prediction and intervention but also establishes the foundation for potential therapeutic modalities (Enciso et al. 2020).

Leveraging high-throughput transcriptomic sequencing technology, this study scrutinized sevoflurane-treated rats, successfully identifying a key gene, Fibroblast Growth Factor 2 (Fgf2), with implications for neurocognition (Zhang et al. 2021). Previous investigations have alluded to the potential association between Fgf2 and anesthesia-induced cognitive impairment in elderly individuals (Zhu et al. 2022). Subsequent research endeavors have shed light on the role of Fgf2 in influencing tau protein phosphorylation, a process intimately tied to various neurodegenerative conditions, including Alzheimer’s disease (Scheltens et al. 2021). Furthermore, Fgf2 exercises its effects through the activation of the PI3K/AKT signaling pathway, renowned for its critical role in cell survival, proliferation, and metabolism (Cai et al. 2021). Consequently, Fgf2 emerges as a central molecular mediator bridging the gap between anesthetics and neurocognitive deficits, offering prospective targets for future therapeutic interventions (Smadja et al. 2021).

This study is primarily geared towards examining the impact of sevoflurane exposure on the Fgf2 gene in aged rats while shedding light on the potential molecular mechanisms at play. Recent research endeavors have illuminated sevoflurane’s capacity to induce an upregulation of Fgf2, subsequently impacting the PI3K/AKT/Gsk3b signaling pathway and leading to heightened tau protein phosphorylation. This intricate sequence of molecular events has showcased a significant correlation with cognitive impairments witnessed in the rat model, furnishing invaluable insights into the intricate interplay between anesthetics and neurodegenerative disorders. These insights may lay the foundation for innovative therapeutic strategies in the future.

## Materials and methods

### Transcriptome datasets related to sevoflurane anesthesia in rats from public databases

Transcriptome datasets related to sevoflurane anesthesia in aged rats were obtained from a public database. Specifically, we retrieved data from 18-month-old rat hippocampal tissue samples available in the Gene Expression Omnibus (GEO) database (http://www.ncbi.nlm.nih.gov/geo/) with the Accession Number GSE141242 and Platform GPL22388. This dataset included three rats subjected to oxygen treatment as the control group and three rats exposed to 2.5% sevoflurane anesthesia for a duration of 4 h as the treatment group. These microarray data were utilized for the identification of differentially expressed genes (DEGs) and subsequent GO enrichment analysis.

### Differential expression gene analysis

Data was obtained from the GEO database (dataset GSE141242), and genes with an absolute log fold change (|logFC|) greater than 0.5 and a P-value less than 0.05 were filtered. This selection process was performed using the “limma” package in R software (Ritchie et al. 2015). The resulting Differentially Expressed Genes (DEGs) were further utilized for generating heatmaps and volcano plots, which were implemented using the “heatmap” package and the “ggplot2” package in R software, respectively.

A GO enrichment analysis was performed on 128 DEGs using R software, revealing enriched biological processes and molecular functions. Next, these DEGs were subjected to protein-protein interaction analysis by importing them into the String database (https://cn.string-db.org/cgi/) and GeneMANIA (http://genemania.org/). To further investigate the signaling pathways associated with protein phosphorylation and neurofibrillary tangles, relevant datasets were retrieved from GeneCards and intersected with the 128 DEGs. A Venn diagram was constructed using the Venn diagram tool provided by Xiantao Academic to visualize the overlapping genes. Finally, the significance of differential expression among core genes was statistically assessed using the Welch t-test method.

### Construction of the rat model for neurotoxicity induced by sevoflurane

Wild-type rats were procured from Beijing Weitong Lihua Experimental Animal Technology Co., Ltd. (Beijing, China), with a total of 77 rats acquired, comprising 14 rats at 3 months of age, 14 rats at 12 months of age, and 49 rats at 18 months of age. These rats were maintained under controlled environmental conditions, with a temperature range of 20–24 °C, humidity maintained at 40–60%, and a 12-hour light-dark cycle. They were provided with ad libitum access to food and water. Rats of 3, 12, and 18 months of age were selected and randomly allocated into age-specific groups. Each age group was further subdivided into two groups: the Control group (untreated) and the Sevoflurane (Sevo) group (exposed to 2.5% sevoflurane, procured from Weikqi Biological Technology Co., Ltd., Sichuan, China). Each group consisted of 7 rats. A custom-built sevoflurane anesthesia chamber (50 cm × 30 cm × 30 cm) was utilized, with an airflow rate of 1.5 L/min. The chamber was submerged in a 37 °C water bath and featured two ventilation ports: one connected to the anesthesia pump and the other linked to a gas monitor (Tian et al. 2018). Sevoflurane anesthesia was induced and maintained for 4 h to establish the sevoflurane neurotoxicity rat model (Wang et al. 2020a). Behavioral testing was conducted 1 h post-anesthesia (Shen et al. 2021).

The experimental procedures and group assignments for Fgf2 silencing and Gsk3b activation were conducted as follows: Lentiviruses for Fgf2 silencing were packaged using the core plasmid pLL3.7-Fgf2, which contains the gene silencing sequence, along with helper plasmids (psPAX2 and pMD2.G). Lentivirus packaging services were provided by Shenggong Biotechnology (Shanghai, China). APN/AKT-IN-1, known as an AKT inhibitor that effectively suppresses Gsk3b phosphorylation, thereby activating Gsk3b (Liu et al. 2021a), was utilized as a Gsk3b activator. Rats were subjected to gene silencing and overexpression via bilateral injection techniques. The sham group received an equivalent dose of empty lentivirus vector or DMSO. Eighteen-month-aged rats were selected for the experiments. Prior to commencing the study, the rats were acclimatized to their surroundings under specific conditions for 2 to 3 days. Subsequently, the rats were anesthetized and placed in a stereotaxic apparatus for intracerebral stereotactic injection of the viral solution. The viral solution, containing the lentivirus stock solution (MOI = 100; titer of 2 × 10^8^ ifu/mL) and APN/AKT-IN-1 (10 µM/µL), was injected into the hippocampal CA3 region via bilateral injections using a stereotaxic apparatus. The injection coordinates were as follows: at the intersection of the coronal and sagittal sutures (−4.3 mm posterior; ±2.5 mm lateral; −3.0 mm dorsal), and the injection rate was set at 0.5 µL/min. The needle was left in place for 5 min, and a total volume of 2 µL was injected. Post-surgery, the rats were allowed to recover on a heated blanket and were placed in clean cages for observation (Fang et al. 2013; Chen et al. 2021a; Zhang et al. 2018a; Wang et al. 2022). Rat brain tissues were collected using the perfusion method, with subsequent separation into the cortex and hippocampus for further experimentation (Karamanavi et al. 2022). Random number tables were used to assign mice into groups, and researchers were blinded to the group assignments during animal experiments (Wang et al. 2022). The animal validation experiment groups included the Control group, Sevo group (sevoflurane anesthesia treatment group), Sevo + sh-NC + DMSO group (sevoflurane anesthesia + Fgf2 silencing control + Gsk3b activation treatment control), Sevo + sh-Fgf2 + DMSO group (sevoflurane anesthesia + silencing Fgf2 + Gsk3b activation treatment control), and Sevo + sh-Fgf2 + act-Gsk3b group (sevoflurane anesthesia + silencing Fgf2 + Gsk3b activation treatment group). All animal experiments were conducted in triplicate, and the experimental protocol and animal usage plan were approved by the animal ethics committee of Cancer Hospital of China Medical University, Liaoning Cancer Hospital and Institute.

### Morris water maze (MWM) test

The Morris water maze test was conducted on the rats one hour following the administration of sevoflurane anesthesia (Shen et al. 2021). During the experiment, the rats were not subjected to anesthesia. The experimental setup utilized a circular water tank with a 120 cm diameter and a water depth of 30 cm for rat training and testing. The tank was equipped with cardinal points (north, south, east, and west quadrants) as additional maze cues (Xinruan Information Technology Co., Ltd., XR-SM101, Shanghai, China). The MWM apparatus and the signal acquisition and processing system were located in a soundproofed laboratory. The experiment comprised two phases: a visible platform phase lasting 5 days and a hidden platform phase also spanning 5 days. Each rat underwent three daily trials with a 15-minute interval between trials. The maximum duration allowed for each trial was 60 s. In cases where rats failed to reach the platform within the allotted time, manual guidance to the platform was provided, and escape latency data for the hidden platform test were recorded. Following the completion of the 5-day visible platform test, a 5-day hidden platform test was conducted. Rats were placed into the water maze from the quadrant adjacent to their original platform location, and their time spent and movement trajectory to reach the platform were recorded. This experiment was repeated three times. Escape latency and movement distance data were collected and analyzed using the VisuTrack animal behavior analysis system (XR-VT101, Xinrui Information Technology Co., Ltd., Shanghai, China) (Liao et al. 2022; Tian et al. 2019).

### Open field testing

The open field test serves as a primary method for observing various rat behaviors upon release into an unenclosed environment. It allows for the observation of spontaneous activities in rats, providing insights into the cognitive processes of experimental animals. The experimental setup consisted of a square enclosure made of transparent organic glass with brown walls. Positioned above the experimental platform was a video tracking system, and rats were introduced into the enclosure from the same starting point. Data recorded included the time at which the rats initiated movement upon entering the open field and the cumulative distance they traversed within a five-minute period. Post-experiment cleaning of the equipment at the site was carried out to prevent the generation of any abnormal odors (Zhang et al. 2018b).

### Novel object recognition test

The novel object recognition test comprised specific procedural steps as follows: On the initial day, the rat was gently introduced into the testing chamber and allowed to remain there for a duration of 5 min. Subsequently, on the following day, the rat was gently placed into the test box along with two identical objects. On the third day, the rat was gently positioned at the periphery of the experimental box; however, one of the objects was replaced with a novel object. During the course of the experiment, the exploration time of the rats on each object was recorded whenever the rats made direct contact with the objects using their mouth, forehead, or nose. To calculate the exploration index, the time spent exploring the novel object was divided by the total time expended investigating both objects (Cai et al. 2018; Zhou et al. 2020).

### Primary culture of rat cortical neurons and construction of Sevo neuronal cell model

Embryonic rats, aged 16 to 18 days, were procured from anesthetized pregnant rats, provided by our institution’s animal facility, for the isolation of cortical neurons. The procedure involved the separation of the cerebral cortex, removal of the meninges and blood vessels, followed by mincing the tissue into small fragments. These fragments were subjected to enzymatic digestion using 0.25% trypsin (Cat# 25200-072, Thermo Fisher Scientific, Waltham, MA, USA) at a temperature of 37 °C for a duration of 20 min, after which they were gently triturated. The resulting dispersed cells were then individually placed into culture vessels, including 6-well plates, 96-well plates, and cover glass slides (24 mm×24 mm), all of which had been pre-coated with polylysine at a concentration of 100 µg/ml. These cells were cultivated in neural medium at a controlled environment of 37 °C with 5% CO_2_. The neural medium was formulated with 2% B27 supplement (Cat# 17504-044, Thermo Fisher Scientific), 0.5 mM L-glutamine (Cat# 35050-061, Thermo Fisher Scientific), and 50 U/ml penicillin/streptomycin (Cat# 15140-122, Thermo Fisher Scientific). The culture medium was replenished 8 h post-seeding and subsequently replaced with half of the medium every alternate day. To confirm the identity of the neurons, they were subjected to staining with class III β-tubulin protein (1:100, ab18207, Abcam, UK) and Hoechst 33342 (14533, Sigma-Aldrich, MO, USA) (Lai et al. 2020; Wang et al. 2022).

Cortical neurons cultured in vitro were exposed to a 3.4% sevoflurane concentration for 5 h to obtain the Sevo neuronal cell model. Control group cells were cultured in the complete medium under normal conditions (Xu et al. 2018).

### Cell transfection

To generate Fgf2-silenced cortical neurons, a lentivirus-mediated transfection approach was employed, using the sequences indicated in Table S1 for knockdown. The services of plasmid construction and lentivirus packaging were outsourced to Sengong Biotech in Shanghai, China. The plasmids and lentiviruses utilized were tailored for compatibility with the rat model. A plasmid harboring a single luciferase reporter gene was co-transfected with the requisite helper plasmid into 293T cells (ATCC, CRL-3216, USA). Following validation, amplification, and purification processes, the packaged lentiviruses were obtained. For the lentivirus-mediated cell transfection, 5 × 10^5^ cells were seeded into a 6-well plate. Once cell density reached 70–90%, the medium was supplemented with the appropriate amount of packaged lentivirus (MOI = 10, with a working titer of approximately 5 × 10^6^ TU/mL) and 5 µg/mL polybrene (Merck, TR-1003, USA) for transfection. After 4 h of transfection, an equal volume of medium was added to dilute the polybrene. Subsequently, fresh medium replaced the transfection medium after 24 h. Observations on the transfection status, as indicated by the luciferase reporter gene, were made 48 h post-transfection. Cortical neuron cells were constructed with Gsk3b activation using APN/AKT-IN-1. The cells were treated with a concentration of 10 µM for a duration of 6 hours (Liu et al. 2021a). In vitro cell validation experiments were categorized into the following groups: Control group, Sevo group (sevoflurane anesthesia treatment group), Sevo + sh-NC group (sevoflurane anesthesia + Fgf2 silence control group), Sevo + sh-Fgf2 group (sevoflurane anesthesia + Fgf2 silence group), Sevo + sh-NC + DMSO group (sevoflurane anesthesia + Fgf2 silence control + Gsk3b activation treatment control group), Sevo + sh-Fgf2 + DMSO group (sevoflurane anesthesia + Fgf2 silence + Gsk3b activation treatment control group), Sevo + sh-Fgf2 + act-Gsk3b group (sevoflurane anesthesia + Fgf2 silence + Gsk3b activation treatment group). The cell-based experiments conducted in this study were replicated three times.

### Immunofluorescence staining and immunohistochemistry

After culturing the neurons for 48 h, 4% paraformaldehyde was added and allowed to fix at room temperature for 15–20 min. The cells were then washed with PBS-0.1% Triton and treated with PBS-0.5% Triton for 5–10 min, followed by another wash with PBS-0.1% Triton. Blocking was performed with 5% BSA for 1 h, followed by overnight incubation with the primary antibody at 4 °C. After washing, the cells were incubated with the secondary antibody at room temperature for 1 h, and then observed under a confocal microscope (Wang et al. 2022).

For tissue samples, fixation was done with 4% paraformaldehyde, followed by sucrose gradient dehydration, embedding in OCT, and tissue sectioning. Immunofluorescence co-staining was performed to detect P-tau co-expression in hippocampal tissue. After rehydration of the sections, blocking was done with 2% BSA. The sections were then incubated with the appropriate primary antibody at room temperature overnight. After washing, immunofluorescent staining was performed using DAPI (Thermo Fisher, 62248, USA) for 10 min, followed by sealing the sections with an anti-quenching mounting medium (Tian et al. 2022).

For immunohistochemistry, tissue sections were treated with biotin-labeled secondary antibody and incubated at room temperature or 37 °C for 30 min to 1 h. PBS was used for rinsing (3 min, 5 times). DAB staining was performed for 5–10 min, followed by thorough rinsing with tap water for 10 min. Counterstaining was done with hematoxylin, and after rinsing with tap water until clear, the sections were treated with 1% hydrochloric acid for 3 s, rinsed with tap water 5 times, and then immersed in lithium carbonate (bluing solution) for 2 s. The sections were further rinsed for 15 min with water and dehydrated with 85% ethanol for 5 min, followed by 95% ethanol and 100% ethanol for 5 min each. After two washes in xylene (10 min each), the sections were air-dried with xylene in a fume hood and mounted with neutral gum. Neurofibrillary tangle density in hippocampal tissue was quantitatively analyzed using the highest pathological density region (Josephs et al. 2015; Li et al. 2023).

Fluorescent staining results were observed and saved using a laser scanning confocal microscope (Leica, STELLARIS 5, Germany), and Image pro plus software was used for semi-quantitative analysis of the results (Daniel et al. 2021).

The primary antibody used for immunofluorescence was P-tau (1:100, Thermo Fisher, 44-750G, Rabbit, USA), and the secondary antibody was Rabbit anti-Rat IgG (H + L) Cross-Adsorbed Secondary Antibody, Alexa Fluor™ 594 (1:1000, Thermo Fisher, A-21,211, USA).

### RT-qPCR

Total RNA was extracted from the specimens using Trizol reagent (Thermo Fisher, 16096020, USA). Subsequently, reverse transcription was carried out employing a reverse transcriptase kit (Takara, RR047A, Japan) to generate complementary DNA (cDNA). The One-Step TB Green® PrimeScript™ RT-PCR Kit (Takara, RR066A, Japan) was utilized to establish the reaction system, and RT-qPCR reactions were conducted by placing the samples into a real-time fluorescence quantitative PCR instrument (Thermo Fisher, ABI 7500, USA). Gapdh served as the internal reference gene. The PCR program was designed as follows: Initial pre-denaturation at 95 °C for 30 s, followed by a cycling process comprising denaturation at 95 °C for 5 s and annealing at 60 °C for 30 s. This cycle was repeated 40 times. Subsequently, an extension step was performed at 95 °C for 15 s, followed by another extension at 60 °C for 60 s. Finally, a further extension was conducted at 90 °C for 15 s, and the amplification curve was generated. All RT-qPCR experiments were conducted in triplicate. Please refer to Table S2 for the primer sequences. To represent the fold change in the expression of the target gene between the experimental group and the control group, the 2-ΔΔCt method was employed. The formula for this method is ΔΔCT = ΔCt test - ΔCt control, with ΔCt = Ct target - Ct reference, where Ct signifies the number of amplification cycles necessary for the real-time fluorescence intensity of the reaction to reach a predetermined threshold. This experiment was repeated three times (Tian et al. 2022).

### Western blot

Total protein was extracted from the samples employing the protein extraction kit (Bestbio, BB3101, Shanghai, China), and the protein concentration was determined utilizing the BCA assay kit (Beyotime, P0012S, Shanghai, China). Subsequently, a 10% SDS-PAGE gel (Bio-Rad, P0012A, Shanghai, China) was prepared. In each well, 50 µg of protein samples were loaded, and constant voltage electrophoresis was conducted for 2 h, ranging from 80 to 120 V. The protein was then transferred onto a PVDF membrane (Merck, IPVH00010, Germany) utilizing a constant current of 250 mA for 90 min. The PVDF membrane was incubated with TBST containing 5% skimmed milk powder at room temperature for 2 h. Subsequently, the blocking solution was discarded, and the membrane was washed with TBST for 10 min. After overnight incubation with the primary antibody at 4 °C (antibody details in Table S3, Thermo Fisher, Abcam), the membrane underwent three 10-minute washes with TBST. It was then incubated with Goat anti-rabbit IgG (Abcam, ab6721, UK) or Goat anti-mouse IgG (Abcam, ab6789, UK) diluted to a 1:2000 concentration at room temperature, with horseradish peroxidase conjugation for 1 h. This was followed by three 10-minute washes with PBST. For color development, the ECL reagent (Bi Yun Tian, P0018FS, Shanghai, China) was employed, and the membrane was placed in a dark box for exposure and development. Each sample experiment was repeated three times (Chen et al. 2022).

### Cell apoptosis detection

Apoptosis in primary rat cortical neurons was assessed utilizing the Annexin V-FITC/PI assay kit (C1062L, Biyun Tian, Shanghai, China). Initially, cells were seeded into a 6-well plate at a density of 1 × 106 cells per well. Following cell collection, cells were resuspended in 195 µL of Annexin V-FITC binding solution, to which 5 µL of Annexin V/FITC and 10 µL of PI were added. The mixture was incubated at room temperature in darkness for 15 min. Subsequently, flow cytometry analysis was conducted within 20 min to determine the apoptosis rate. The apoptosis rate was determined as the combined proportion of apoptotic cells found in the Q2-UR (upper right) and Q3-LR (lower right) quadrants (Chen et al. 2021b).

### Detection of oxidative stress-related indicators

Following euthanasia, the rats underwent dissection, and their brains were partitioned into contralateral and ipsilateral hemispheres to facilitate the isolation of the ipsilateral cortical tissue. The cerebral cortex tissue was immersed in physiological saline (in a 1:9 ratio) within an ice bath at 4 °C. Subsequently, high-speed homogenization at 4 °C and 4000 r/min for 10 min was employed, followed by centrifugation. The resulting clear upper liquid was transferred into containers and stored at −20 °C (Xu et al. 2021). Regarding cell sample collection, cells were initially cultured in a 6-well plate and subjected to the appropriate treatments. After incubation, PBS was utilized for a single wash, followed by cell collection through centrifugation. Subsequently, the supernatant was obtained by lysing the cells with lysis buffer and subsequent centrifugation (Lin et al. 2021). The assessment of lipid peroxidation was carried out in accordance with the instructions provided with the assay kit for thiobarbituric acid reactive substances (TBARS), which was used to quantify lipid peroxidation. The evaluation of lipid peroxidation product levels was expressed in terms of malondialdehyde (MDA) equivalents. Additionally, the measurement of glutathione (GSH) levels was conducted using the GSH detection kit (ab239727, Abcam, UK) (Lin et al. 2020a).

### ROS detection

The experimental procedures were carried out in strict accordance with the instructions provided by the reactive oxygen species assay kit (S0033S, Bi Yun Tian, Shanghai, China). Specifically, 2,7-dichlorodihydrofluorescein diacetate (DCFH-DA), a fluorescent probe, was introduced into the tissue supernatant at a concentration of 10 mol/L (190 µL). Subsequently, the samples were incubated in darkness at 37 °C for a duration of 30 min. The quantification of green fluorescence intensity was accomplished using an automated fluorescence microplate reader (model 5200110, Thermo Fisher, MA, USA) equipped with an excitation wavelength of 488 nm and an emission wavelength of 530 nm. The level of reactive oxygen species (ROS) within the tissue was determined as the fluorescence intensity ratio relative to the protein concentration (He et al. 2019; Li et al. 2021). For the assessment of ROS production within live cells, primary cortical neurons that had been seeded onto coverslips underwent a rinsing step with pre-warmed PBS. Subsequently, they were subjected to incubation with 3 µM of dihydroethidium (DHE) dye (D7008, Sigma-Aldrich, MO, USA) at 37 °C for 30 min. Following this incubation period, thorough washing was performed, and the samples were examined under a confocal microscope utilizing a 20× objective lens (LSM 750, Carl Zeiss, Goettingen, Germany). The quantification of DHE fluorescence intensity was executed using ImageJ software. The ROS levels were calculated based on the average fluorescence intensity across six randomly selected fields in each experiment, and these values were expressed as multiples of the control intensity (Lai et al. 2020).

### Thioflavin T staining

Thioflavin T staining method was employed to quantify the levels of neurofibrillary tangles (NFTs). Tissue sections were brought to room temperature and washed three times for 5 min each in tbs (A510025, Sangon Biotech). Then, the tissue sections were immersed in a light-protected solution of 0.0125% Thioflavin T in ethanol (HY-D0972, MedChemExpress) for 8 min. Subsequently, the tissue sections were washed three times for 5 min each in 50% ethanol, followed by one wash in TBS for 5 min. After drying the tissue sections in a light-protected manner, glycerin was used for sealing. The staining results were observed and photographed under a fluorescence microscope (Olympus, Tokyo, Japan).

### Statistical analysis

This study utilized software versions including R 4.2.1, with RStudio 4.2.1 as the integrated development environment for R software compilation. File processing was carried out using Perl language, specifically Perl 5.30.0. Additionally, Cytoscape 3.7.2 and SPSS statistical software version 21.0 (IBM SPSS Statistics, Chicago, USA) were employed. Quantitative data were presented as mean ± standard deviation, and independent sample t-tests were employed for comparisons between two groups. Comparisons of data among different time points within each group were conducted through repeated measures analysis of variance (ANOVA), with post hoc testing performed using the Bonferroni method. A significance level of *P* < 0.05 was considered to indicate statistical significance.

## Results

### Age-dependent cognitive impairments in rats induced by sevoflurane exposure: behavioral insights from water maze and open-field tests

Postoperative cognitive dysfunction (POCD) is a common but underestimated complication, especially in older people. However, its pathogenesis has not been reported, and it may be related to multiple factors such as surgical stress, anesthesia drugs, pain, inflammatory response, hypoxia, and postoperative complications (Kotekar et al. 2018; Lin et al. 2020b). According to literature reports, Sevoflurane (Sevo) has a particular impact on POCD in neurodegenerative diseases (Xu et al. 2014; Ni et al. 2015). However, the effects of sevoflurane on gene expression patterns and regulatory networks during general anesthesia remain to be elucidated. Due to the induction of spatial learning and memory impairments in rats, sevoflurane has been found effective (Lamberty and Gower 1991). We established a rat model of sevoflurane-induced developmental neurotoxicity to explore the specific molecular mechanisms of sevoflurane on cognitive impairment.

**Fig. 1:**
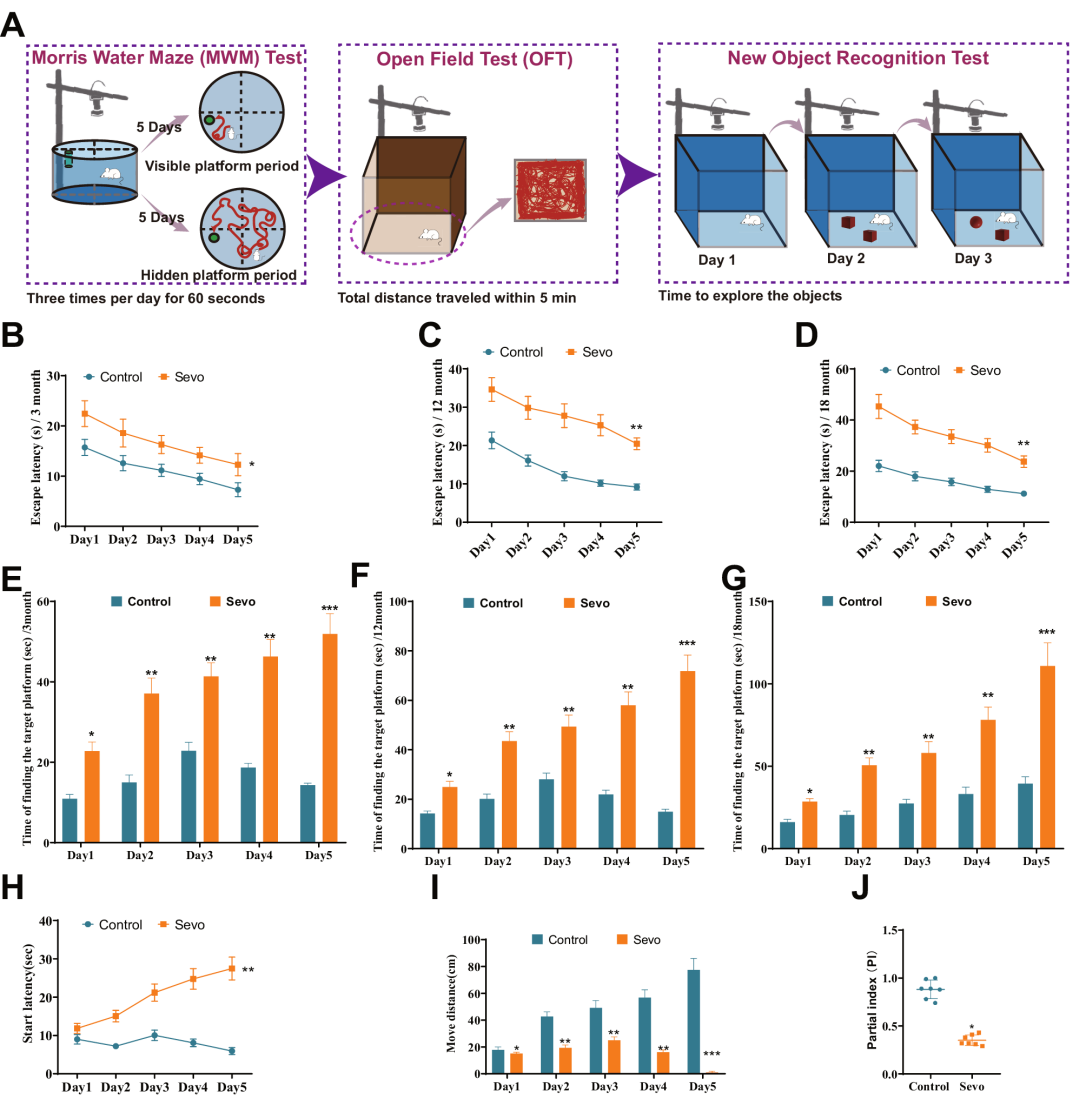
Effects of sevoflurane exposure on rats’ learning, cognition, and motor ability. *Note*: (**A**) Behavioral Experiment Design and Timeline; (**B**–**D**) Line graphs showing the changes in escape latency during different days in the water maze experiment for rats in different treatment groups at 3 months (Fig. B), 12 months (Fig. C), and 18 months (Fig. D); (**D**–**F**) Bar graphs showing the time required for rats in different treatment groups at 3 months (Fig. E), 12 months (Fig. F), and 18 months (Fig. G) to find the target platform on different days in the water maze experiment; (**H**) Open field test measuring the time for 18-month-aged rats in different treatment groups to initiate movement; (**I**) Open field test measuring the total distance traveled by 18-month-aged rats in different treatment groups; (**J**) Index of novel object recognition in the open field test. *P* < 0.05, *P* < 0.01, **P < 0.001, all significances are compared between the Sevo and Control groups; each group consisted of 7 rats

To investigate the effects of sevoflurane on cognitive impairment in rats of different age groups, we designed experiments using a water maze and an open-field test. We recorded and analyzed the behavioral and cognitive indicators of the tested rats. This study used rats at 3 months, 12 months, and 18 months of age, corresponding to the developmental, mature, and aging stages in humans (Andreollo et al. 2012). A series of behavioral experiments was conducted on rats (Fig. 1A). The Morris water maze experiment results showed that compared with the control group, the escape latency of rats induced by sevoflurane was increased in different age groups. The difference in escape latency was particularly in 12-month-old and 18-month-aged rats (Fig. 1B–D). Rats searching for hidden platforms showed that rats induced with sevoflurane took longer to find the hidden platform, and the time spent also increased with the age of the rats (Fig. 1E–G). The above results indicate that sevoflurane induces learning impairments in rats, and the impact on learning ability in aged rats is more significant than in young rats.

Subsequently, we chose 18-month-aged rats for the open-field experiment. The results showed that, compared with the control group, the time for aged rats induced by sevoflurane to start moving in the open field was increased (Fig. 1H), and the distance traveled in the open field after entry was reduced (Fig. 1I). In the novel object recognition test, the frequency of exploration of novel objects induced by sevoflurane in rats was decreased (Fig. 1J). The behavioral experiment results indicate that sevoflurane-induced motor and cognitive impairments in rats.

In summary, sevoflurane-induced cognitive impairment in rats, and this effect worsened with increasing age of the rats.

### Transcriptomic analysis reveals Fgf2 as a key regulator in sevoflurane-induced neurocognitive impairment in rat hippocampus

We obtained the rat hippocampus-related transcriptome sequencing (RNA sequencing, RNA-seq) dataset GSE141242 from Gene Expression Omnibus (GEO) and analyzed it. The dataset includes three standard hippocampal tissue samples (GSM4199182, GSM4199183, GSM4199184) and three hippocampal tissue samples from rats induced with 2.5% sevoflurane neurotoxicity (GSM4199185, GSM4199186, GSM4199187).

**Fig. 2:**
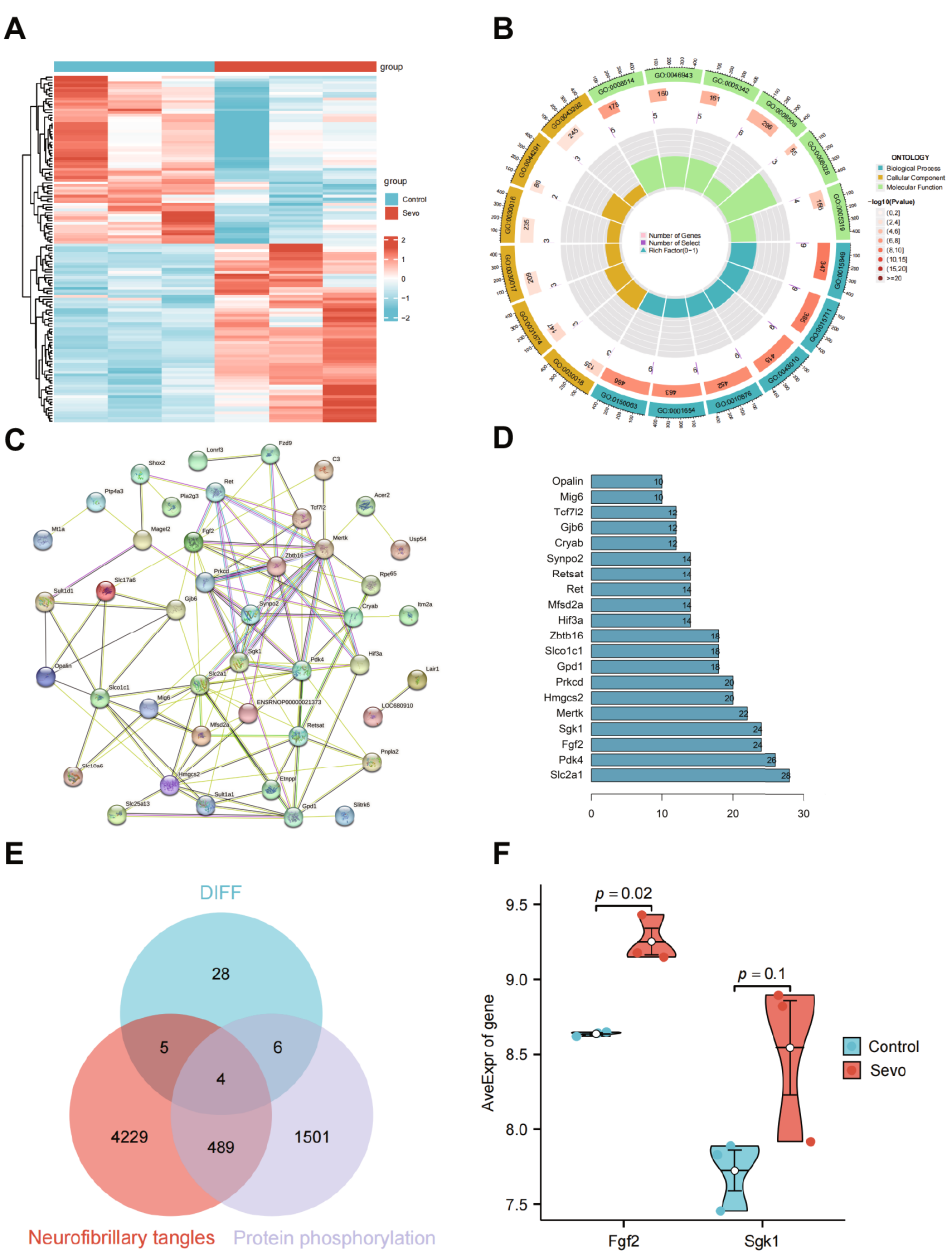
Core factors involved in sevoflurane-induced cognitive impairment identified by bioinformatics analysis. *Note*: (**A**) Heatmap showing the differentially expressed genes (DEGs) between standard control samples (Control group, *n* = 3) and sevoflurane-induced neurotoxicity rat samples (Sevo group, *n* = 3) in GSE141242 (LogFC > 0.5, P value < 0.05). (**B**) Functional enrichment analysis results of the DEGs at the biological process (BP), cellular component (CC), and molecular function (MF) levels. (**C**) Protein-protein interaction (PPI) network was constructed for the 128 encoded proteins of the DEGs identified in GSE141242. (**D**) Top 20 genes ranked by the number of connections in the PPI network. (**E**) The intersection between 43 DEGs (DIFF) identified in GSE141242 and genes associated with neurofibrillary tangles and protein phosphorylation. (**F**) Analysis of the differential expression of critical genes Fgf2 and Sgk1 using the Welch to test statistical method

Through integration and differential analysis, we identified 128 DEGs, which comprised 66 upregulated genes and 62 downregulated genes (Fig. 2A). Furthermore, GO enrichment analysis was performed on the selected 128 DEGs, and the results indicated that these DEGs were mainly enriched in biological processes such as lipid localization, camera-type eye development, and organic acid transport. In terms of molecular functions, they were enriched in actin filament bundle, contractile fiber, and cell-cell contact zone. Additionally, they were found to be involved in signaling pathways, including transmembrane transporter, lipid transporter activity, and organic anion transmembrane (Fig. 2B). We imported the proteins coded by these DEGs into the String database for protein-protein interaction analysis and constructed a gene interaction network diagram (Fig. 2C). The number of network connection nodes for each gene was also counted (Fig. 2D). The results showed that the top five genes in terms of node numbers were Slc2a1, Pdk4, Fgf2, Sgk1, and Mertk. These genes played essential roles as critical factors in the network.

Neurofibrillary tangles are one of the abnormal structures found in the brains of Alzheimer’s disease patients, primarily composed of hyperphosphorylated tau protein (Gómez-Ramos et al. 2004; Geschwind 2003). Therefore, abnormal phosphorylation of tau protein and the formation of neurofibrillary tangles are essential factors in the development of neurocognitive disorders. To further identify the core genes associated with sevoflurane-induced cognitive impairment in rats, we identified 43 differential proteins (DIFF) through the String online database and constructed a protein-protein interaction network. We then used GeneCards to search for genes related to neurofibrillary tangles and protein phosphorylation in neurons and intersected these genes with the DIFF genes, resulting in four differential genes (Fgf2, Sgk1, Prkcd, and Pnpla2) (Fig. 2E). Fgf2 and Sgk1 are among the top five genes regarding the number of connecting nodes. To further identify the target genes, we analyzed the expression differences of Fgf2 and Sgk1 using the Welch t-test statistical method. The results showed that the expression of Fgf2 was higher, and the difference was more (Fig. 2F).

The above results indicate that Fgf2 is a critical gene that regulates the neurocognitive impairment induced by sevoflurane anesthesia.

### Silencing Fgf2 alleviates sevoflurane-induced neuronal apoptosis, oxidative stress, and neuroinflammation in cultured rat cortical neurons

Studies indicate that the Fgf2 gene is closely associated with Alzheimer’s disease and other neurological and cognitive disorders (Kiyota et al. 2011). According to the expected experimental results of the first two parts of the experimental design, sevoflurane could induce upregulation of the Fgf2 gene in the hippocampal tissue of 18-month-aged rats. To investigate the mechanism of the impact of sevoflurane-induced Fgf2 expression on cognitive impairment, we isolated and cultured primary rat cortical neurons and constructed an in vitro cell model (Sevo model neurons) induced by sevoflurane. By transducing viral vectors to silence Fgf2, we further investigated the effects of Fgf2 on neuronal apoptosis, oxidative stress, and neuroinflammation-related factors in the Sevo model. First, we assessed the morphology and purity of the cultured primary cortical neurons. As shown in Fig. S1, after two days of cultivation, the neurons are smaller, with relatively round cell bodies and shorter neurites (Fig. S1A). Expand and form a network on the 7th day (Fig. S1B). Immunofluorescence staining showed that the cell bodies and ganglia of neurons were stained red with β-III tubulin protein, and approximately 90% of the cells were neurons (Fig. S1C). The above results indicate that the cultured neurons have good purity and could be used for subsequent experiments.

**Fig. 3:**
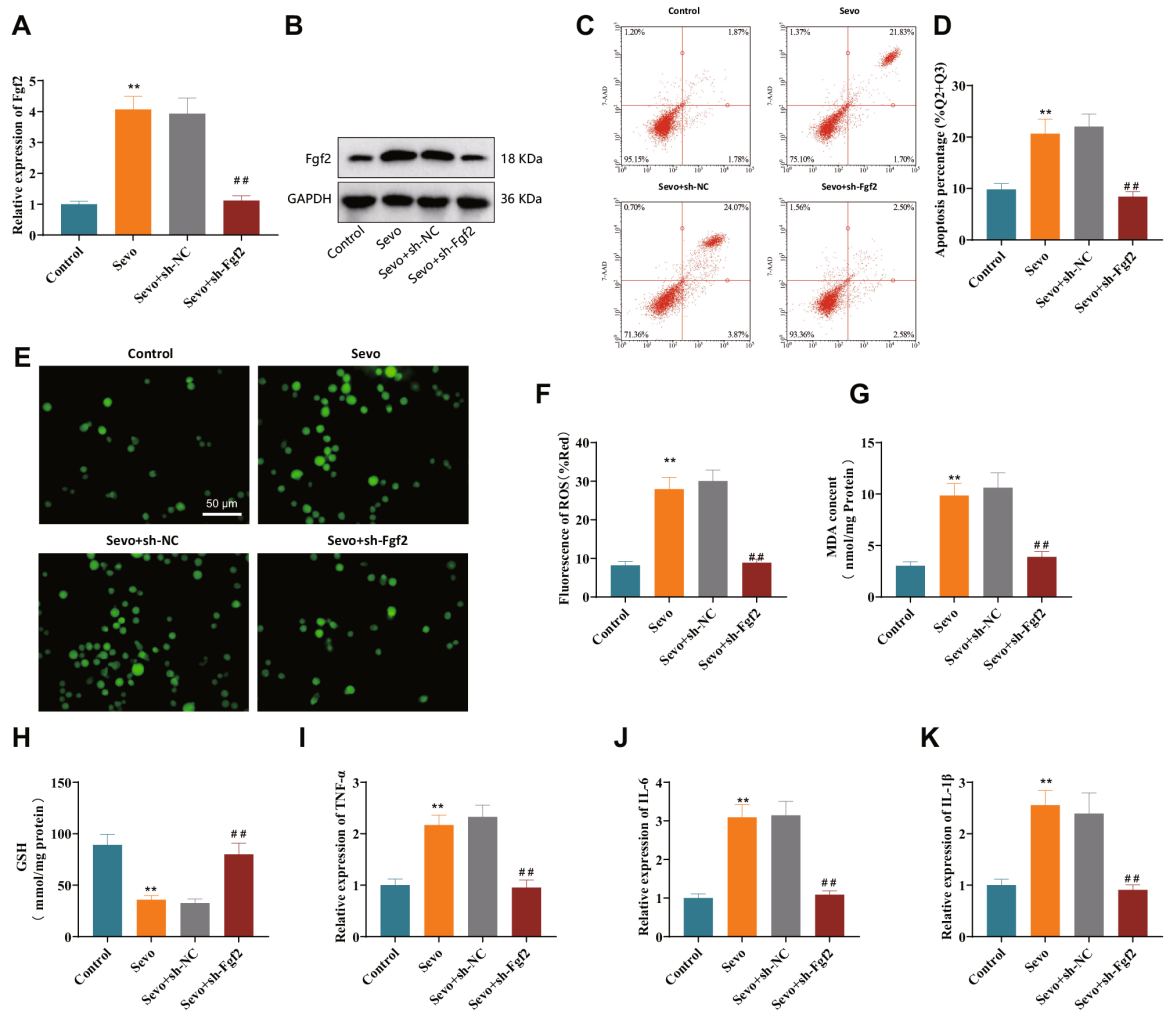
Effects of silencing Fgf2 on neuronal apoptosis, oxidative stress injury, and neuroinflammation in the in vitro sevoflurane model. *Note*: (**A**) RT-qPCR analysis of Fgf2 mRNA expression in sevoflurane model neurons; (**B**) Western blot analysis of Fgf2 protein expression in sevoflurane model neurons; (**C**) Flow cytometry analysis of apoptosis in primary cortical neurons from different groups (Q2 and Q3 quadrants); (**D**) Statistical analysis of the percentage of apoptosis in different groups of primary cortical neurons (Q2 and Q3 quadrants); (**E**–**F**) Generation of reactive oxygen species (ROS) in different groups of primary cortical neurons (green fluorescence represents ROS activity marker, scale bar = 50 μm); (**G**) Measurement of malondialdehyde (MDA) content in different groups of primary cortical neurons using the TBARS assay; (**H**) Measurement of glutathione (GSH) content in different groups of primary cortical neurons using the glutathione assay kit; (**I**–**K**) RT-qPCR analysis of TNF-α, IL-6, and IL-1β expression in different groups of primary cortical neurons. ** represents a difference compared to the Control group or Sevo + sh-NC group (*P* < 0.01), ^##^ represents a difference compared to the Sevo + sh-Fgf2 group (*P* < 0.01), and the cell experiments were repeated 3 times

Furthermore, we used lentivirus transfection to silence the expression of Fgf2 and validated the silencing effect of the two sh-Fgf2 sequences by RT-qPCR and Western blot. Silence efficiency is shown in Fig. S2. We choose sequences that are more efficient in silence for subsequent experiments. Expression of Fgf2 was detected in each treatment group using RT-qPCR and Western blot. The results showed a*n* increase in the expression of Fgf2 in the neurons of the Sevo group compared to the Control group. Compared to the Sevo + sh-NC group, the expression of Fgf2 in neuronal cells decreased in the Sevo + sh-Fgf2 group (Fig. 3A, B).

Furthermore, we employed flow cytometry to assess neuronal apoptosis. The results indicated that neuronal apoptosis increased in the Sevo group compared to the Control group. Compared to the Sevo + sh-NC group, the Sevo + sh-Fgf2 group showed increased neural proliferation and reduced cell apoptosis (Fig. 3C, D). We also used commercial assay kits to assess ROS, MDA, and GSH levels in cells. The results show that compared to the Control group, the levels of ROS and MDA increased in the Sevo group, while GSH levels decreased, indicating that cells were under oxidative stress. Compared with the Sevo + sh-NC group, the Sevo + sh-Fgf2 group showed decreased levels of ROS and MDA, and an increased level of GSH, indicating reduced oxidative stress damage in cells (Fig. 3E–H). Finally, we assessed the expression levels of neuroinflammation-related factors in neuronal cells using RT-qPCR. TNF-α, IL-6, and IL-1β expression increased in the Sevo group compared to the Control group. Compared to the Sevo + sh-NC group, the expression levels of pro-inflammatory cytokines in neuronal cells decreased in the Sevo + sh-Fgf2 group (Fig. 3I–K). The above results indicate that silencing Fgf2 could reduce neuronal apoptosis, and decrease cellular oxidative stress levels and neuroinflammation, thereby alleviating sevoflurane-induced neurocognitive impairments.

### Fgf2 silencing modulates tau phosphorylation via the PI3K/AKT-Gsk3b pathway, reducing neuronal apoptosis and oxidative stress under sevoflurane anesthesia

Tau protein plays a crucial role in the occurrence of neurological disorders. Excessive phosphorylation could lead to the loss of normal neuronal function and subsequently trigger neurodegenerative diseases (Wegmann et al. 2021). Gsk3b (Glycogen Synthase Kinase 3 Beta) plays a vital role in the pathological mechanism of tau protein hyperphosphorylation as a Ser/Thr kinase. It could catalyze and regulate the phosphorylation of Ser 262 and Ser 396/404 sites of tau protein (Hernandez et al. 2013). Upregulation of the Fgf2 gene inhibits the phosphorylation levels of PI3K/AKT. The decrease in the phosphorylation level of PI3K/AKT leads to a reduction in the phosphorylation level of Gsk3b, and the activity of Gsk3b is closely correlated with its phosphorylation level (Ojeda et al. 2011; Hong et al. 2016). Furthermore, the literature reports that Fgf2 could positively regulate the activity of Gsk3b, promoting the phosphorylation of tau protein (Tatebayashi et al. 1999; Tanaka et al. 2000). However, it is currently unclear whether the upregulation of Fgf2 induced by sevoflurane could affect tau protein phosphorylation by regulating Gsk3b.

**Fig. 4:**
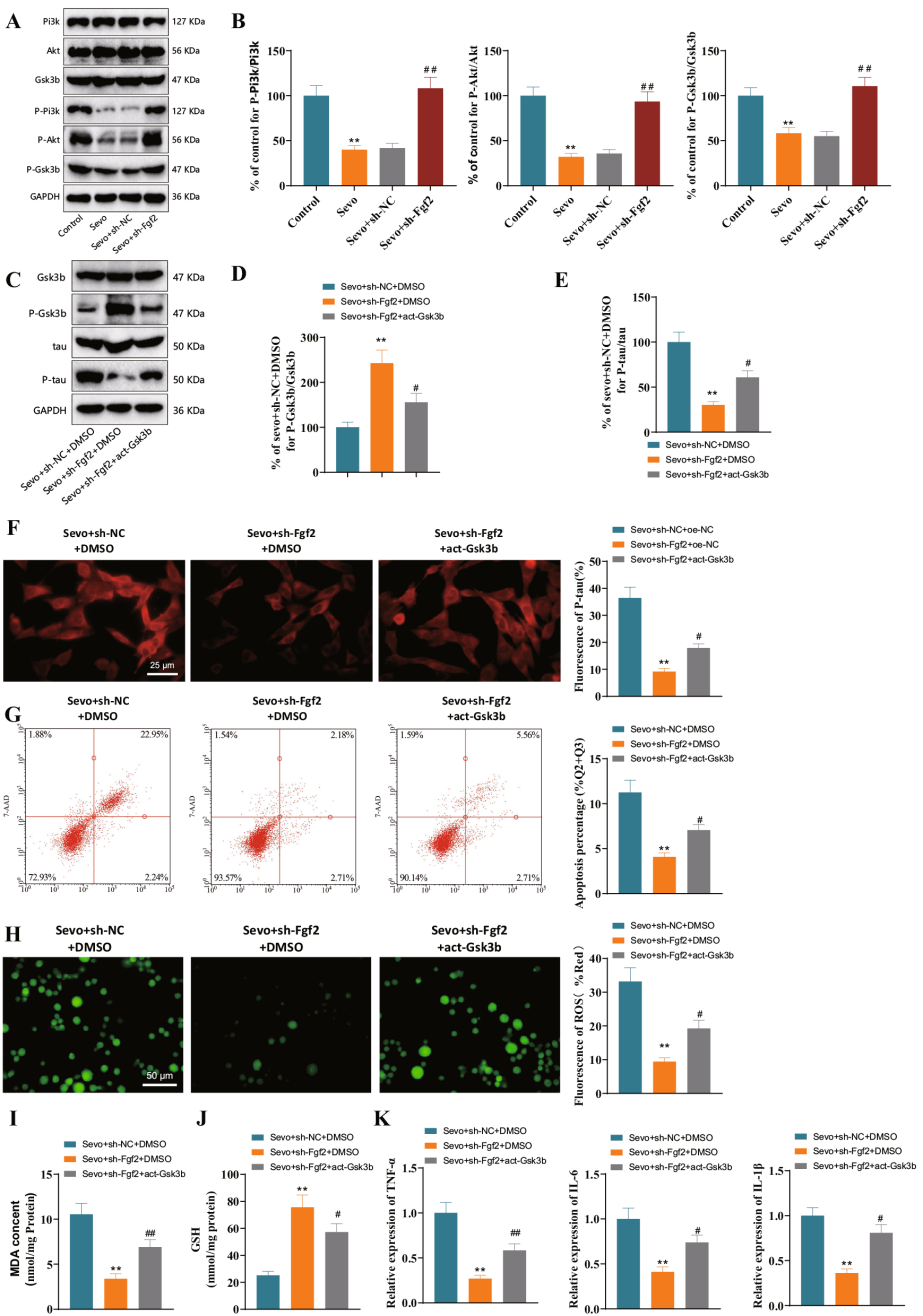
Fgf2 regulates PI3K/AKT/Gsk3b pathway to promote tau protein phosphorylation in Sevo model neurons. *Note*: (**A**) Western blot analysis of protein expression of PI3K, AKT, Gsk3b, P-PI3K, P-AKT, and P-Gsk3b in Sevo model neurons after silencing Fgf2. (**B**) Statistical analysis of protein expression ratios of P-PI3K/PI3K, P-AKT/AKT, P-Gsk3b/Gsk3b in Sevo model neurons after silencing Fgf2. (**C**) Western blot analysis of protein expression of Gsk3b, P-Gsk3b, tau, and P-tau in Sevo model neurons after silencing Fgf2 and Gsk3b activation. (**D**) Statistical analysis of protein expression ratio of P-Gsk3b/Gsk3b. (**E**) Statistical analysis of protein expression ratio of P-tau/tau. (**F**) Immunofluorescence detection of P-tau content in primary cortical neurons of various groups after silencing Fgf2 and Gsk3b activation. (**G**) Flow cytometry analysis of apoptosis and proportion (Q2 and Q3 quadrants) in primary cortical neurons of various groups after silencing Fgf2 and Gsk3b activation. (**H**) Measurement of ROS production in primary cortical neurons of various groups after silencing Fgf2 and Gsk3b activation. (**I**) TBARS assay to detect MDA levels in primary cortical neurons of various groups after silencing Fgf2 and Gsk3b activation. (**J**) Glutathione assay to detect GSH levels in primary cortical neurons of various groups after silencing Fgf2 and Gsk3b activation. (**K**) RT-qPCR analysis of TNF-α, IL-6, and IL-1β expression in primary cortical neurons of various groups after silencing Fgf2 and Gsk3b activation. ** indicates comparison with Control group or Sevo + sh-NC + DMSO group, *P* < 0.01; ^#^ indicates comparison with Sevo + sh-Fgf2 + DMSO group, *P* < 0.05; ^##^ indicates comparison with Sevo + sh-NC group, *P* < 0.01. Cell experiments were repeated three times

To investigate whether Fgf2 could regulate Gsk3b through the PI3K/AKT signaling pathway under the induction conditions of sevoflurane anesthesia, we intervened in the expression of Fgf2 using the lentivirus transfection method. The phosphorylation levels of PI3K, AKT, and Gsk3b were measured by Western blot in different treatment groups. The results showed that compared to the Control group, the phosphorylation levels of PI3K, AKT, and Gsk3b in neurons of the Sevo group decreased. Compared to the Sevo + sh-NC group, the phosphorylation levels of PI3K, AKT, and Gsk3b in neurons of the Sevo + sh-Fgf2 group increased (Fig. 4A, B). The above results indicate that the silence of Fgf2 stimulates the PI3K/AKT signaling pathway, thereby promoting the phosphorylation of Gsk3b and inhibiting its activity.

To further investigate whether Fgf2 could regulate the phosphorylation of tau protein induced by sevoflurane anesthesia, we used lentiviral transfection to intervene in the expression of Fgf2 and used APN/AKT-IN-1 as an activator of Gsk3b to manipulate the activity of Gsk3b. The phosphorylation levels of Gsk3b and tau in different treatment groups were detected by Western blot. The results showed that compared to the Sevo + sh-NC + DMSO group, the phosphorylation level of Gsk3b in neurons increased in the Sevo + sh-Fgf2 + DMSO group, while the phosphorylation level of tau decreased. Compared to the Sevo + sh-Fgf2 + DMSO group, the phosphorylation level of Gsk3b in neurons decreased in the Sevo + sh-Fgf2 + act-Gsk3b group, while the phosphorylation level of tau increased (Fig. 4C–E). Subsequently, we assessed the expression levels of P-tau in neurons of each treatment group using immunofluorescence detection. The results showed that the fluorescence signal of the Sevo + sh-Fgf2 + DMSO group was attenuated compared to the Sevo + sh-NC + DMSO group.

Additionally, the fluorescence signal of the Sevo + sh-Fgf2 + act-Gsk3b group was enhanced compared to the Sevo + sh-Fgf2 + DMSO group (Fig. 4F). The above results indicate that silencing Fgf2 inhibits the activity of Gsk3b, thereby reducing the phosphorylation level of the tau protein. After activation, Gsk3b exhibits increased activity, leading to a*n* elevation in tau phosphorylation levels.

Phosphorylation of Tau protein reduces oxidative stress damage, improves neuroinflammation, and protects neuronal cells (Yang et al. 2022). On this basis, we examined the effects of intervention with Fgf2 and Gsk3b on neuronal apoptosis, cellular oxidative stress levels, and the expression of neuroinflammatory factors. The results of neuronal apoptosis showed that compared with the Sevo + sh-NC + DMSO group, the Sevo + sh-Fgf2 + DMSO group had reduced neuronal apoptosis, while compared with the Sevo + sh-Fgf2 + DMSO group, the Sevo + sh-Fgf2 + act-Gsk3b group had increased neuronal apoptosis (Fig. 4G). We used commercial reagent kits to evaluate the ROS, MDA, and GSH levels in various groups of cells. The results showed that compared to the Sevo + sh-NC + DMSO group, the Sevo + sh-Fgf2 + DMSO group had lower levels of ROS and MDA, while the level of GSH was higher, indicating a reduction in oxidative stress damage to the cells.

In contrast, compared to the Sevo + sh-Fgf2 + DMSO group, the Sevo + sh-Fgf2 + act-Gsk3b group had higher levels of ROS and MDA, while the level of GSH was lower, indicating that the cells were in a state of oxidative stress (Fig. 4H–J). Finally, we detected the expression levels of neuroinflammation-related factors in neuronal cells using RT-qPCR. The results showed that compared to the Sevo + sh-NC + DMSO group, the Sevo + sh-Fgf2 + DMSO group decreased the expression of TNF-α, IL-6, and IL-1β. Furthermore, compared to the Sevo + sh-Fgf2 + DMSO group, the Sevo + sh-Fgf2 + act-Gsk3b group showed an increase in the expression levels of pro-inflammatory factors in neuronal cells (Fig. 4K).

In summary, silencing Fgf2 could decrease the activity of Gsk3b through PI3K/AKT, thereby inhibiting the phosphorylation of tau protein.

### Silencing of Fgf2 alleviates sevoflurane-induced cognitive dysfunction in rats via downregulation of Gsk3b activity: behavioral evidence

To further investigate the effect of Fgf2 on neurocognitive impairment, we established a rat model of sevoflurane-induced neurocognitive impairment. By packaging and injecting lentiviruses, Fgf2 and Gsk3b were disrupted in individual rats, and behavioral and cognitive indices of the different treatment groups of rats were assessed.

**Fig. 5:**
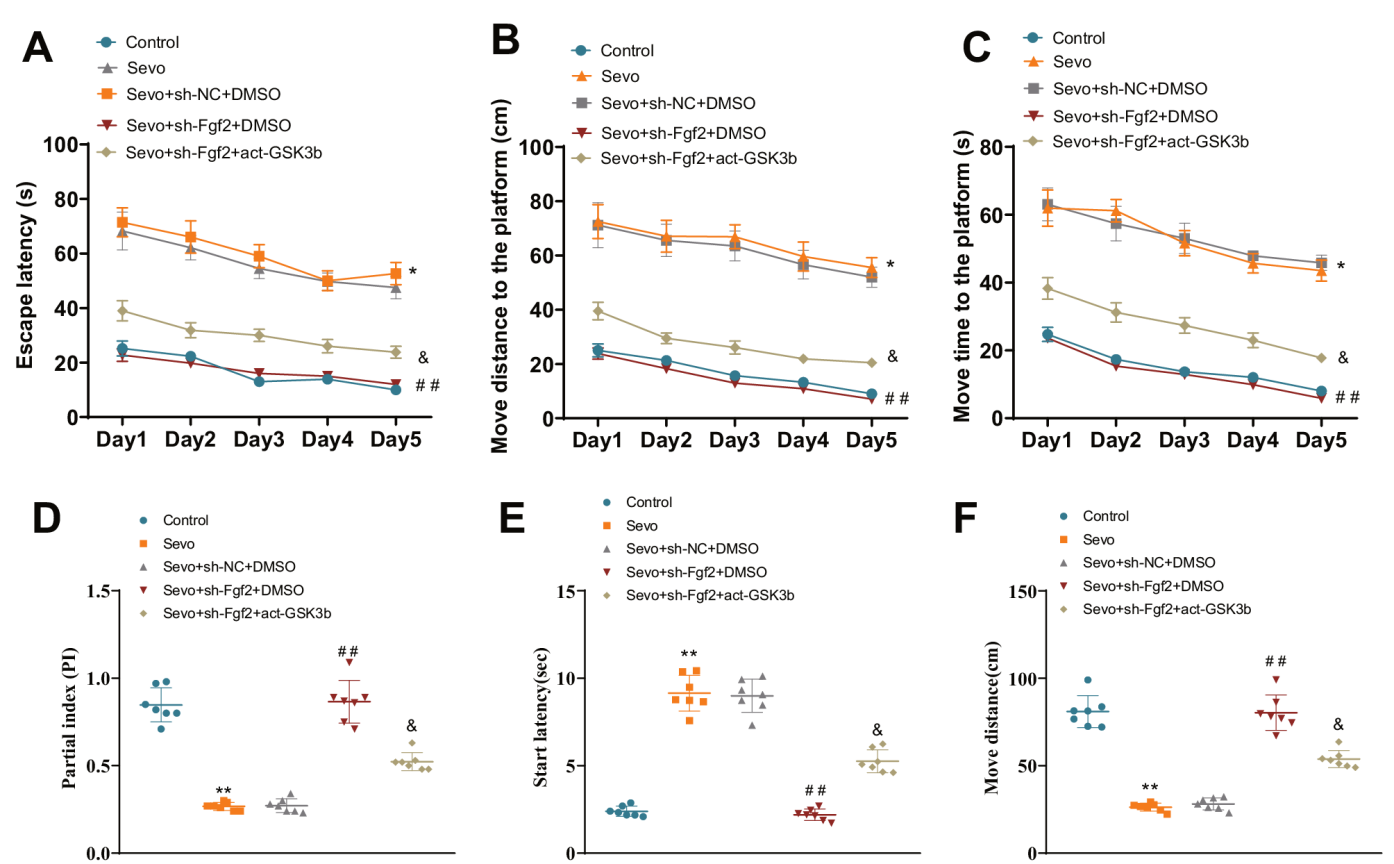
Fgf2 affects motor and learning impairments in aged rats. *Note*: (**A**) Line graph showing the escape latency duration of rats in different treatment groups in the water maze experiment on different days. (**B**) Distance traveled by rats in different treatment groups to reach the specific platform on different days in the water maze experiment. (**C**) Time taken by rats in different treatment groups to reach the specific platform on different days in the water maze experiment. (**D**) Exploration index of rats for the novel object in the open field test. (**E**) Starting time of rat movement in the open field test for different treatment groups. (**F**) Total distance traveled by rats in the open field test for different treatment groups. * indicates comparison with Control group, *P* < 0.05; ** indicates comparison with Control group, *P* < 0.01; ^##^ indicates comparison with Sevo + sh-NC + DMSO group, *P* < 0.01; & indicates comparison with Sevo + sh-Fgf2 + DMSO group, *P* < 0.05. Each group consisted of seven rats

The Morris water maze experiment results showed that compared to the Control group rats, the Sevo group rats had increased latency to escape. Compared to the Sevo + sh-NC + DMSO group, the Sevo + sh-Fgf2 + DMSO group rats had decreased latency to escape. Compared to the Sevo + sh-Fgf2 + DMSO group, the Sevo + sh-Fgf2 + act-Gsk3b group rats had increased latency to escape (Fig. 5A). The results of the movement distance to the platform in the different groups of rats showed that the Sevo group rats had increased movement distance compared to the Control group rats. Compared to the Sevo + sh-NC + DMSO group, the Sevo + sh-Fgf2 + DMSO group rats had decreased movement distance. Compared to the Sevo + sh-Fgf2 + DMSO group, the Sevo + sh-Fgf2 + act-Gsk3b group rats had increased movement distance (Fig. 5B). The results of the time spent to reach the platform in the different groups of rats showed that compared to the Control group rats; the Sevo group rats spent more time. Compared to the Sevo + sh-NC + DMSO group, the Sevo + sh-Fgf2 + DMSO group rats spent less time reaching the platform. Compared to the Sevo + sh-Fgf2 + DMSO group, the Sevo + sh-Fgf2 + act-Gsk3b group rats spent more time (Fig. 5C).

The novel object recognition test showed that the frequency of exploring novel objects in the Sevo group of rats was decreased compared to the Control group. The exploration frequency of rats in the Sevo + sh-Fgf2 + DMSO group was increased compared to the Sevo + sh-NC + DMSO group. The exploration frequency of rats in the Sevo + sh-Fgf2 + act-Gsk3b group was decreased compared to the Sevo + sh-Fgf2 + DMSO group (Fig. 5D).

Open-field experiment results show that the time for locomotion initiation in the Sevo group rats is later than that in the Control group rats, and the distance traveled is reduced. Compared to the Sevo + sh-NC + DMSO group, the Sevo + sh-Fgf2 + DMSO group rats initiate locomotion earlier in the open field, increasing the distance traveled. Compared to the Sevo + sh-Fgf2 + DMSO group, the Sevo + sh-Fgf2 + act-Gsk3b group rats initiate locomotion later in the open field, reducing the distance traveled (Fig. 5E, F).

The above behavioral experiments in mice have shown that silencing Fgf2 could improve cognitive dysfunction induced by sevoflurane in aged rats by downregulating the activity of Gsk3b.

### Silencing of Fgf2 attenuates tau phosphorylation, reduces neurofibrillary tangles, and mitigates sevoflurane-induced neurocognitive impairment via Gsk3b modulation in rat hippocampus

To investigate the specific mechanisms underlying the effects of Fgf2 expression on neurocognitive behaviors such as motor and learning impairments in rats, we performed dissections on rats from each treatment group and assessed the expression of relevant genes, phosphorylation of tau protein, and neuro fiber tangles in the hippocampal tissue.

**Fig. 6:**
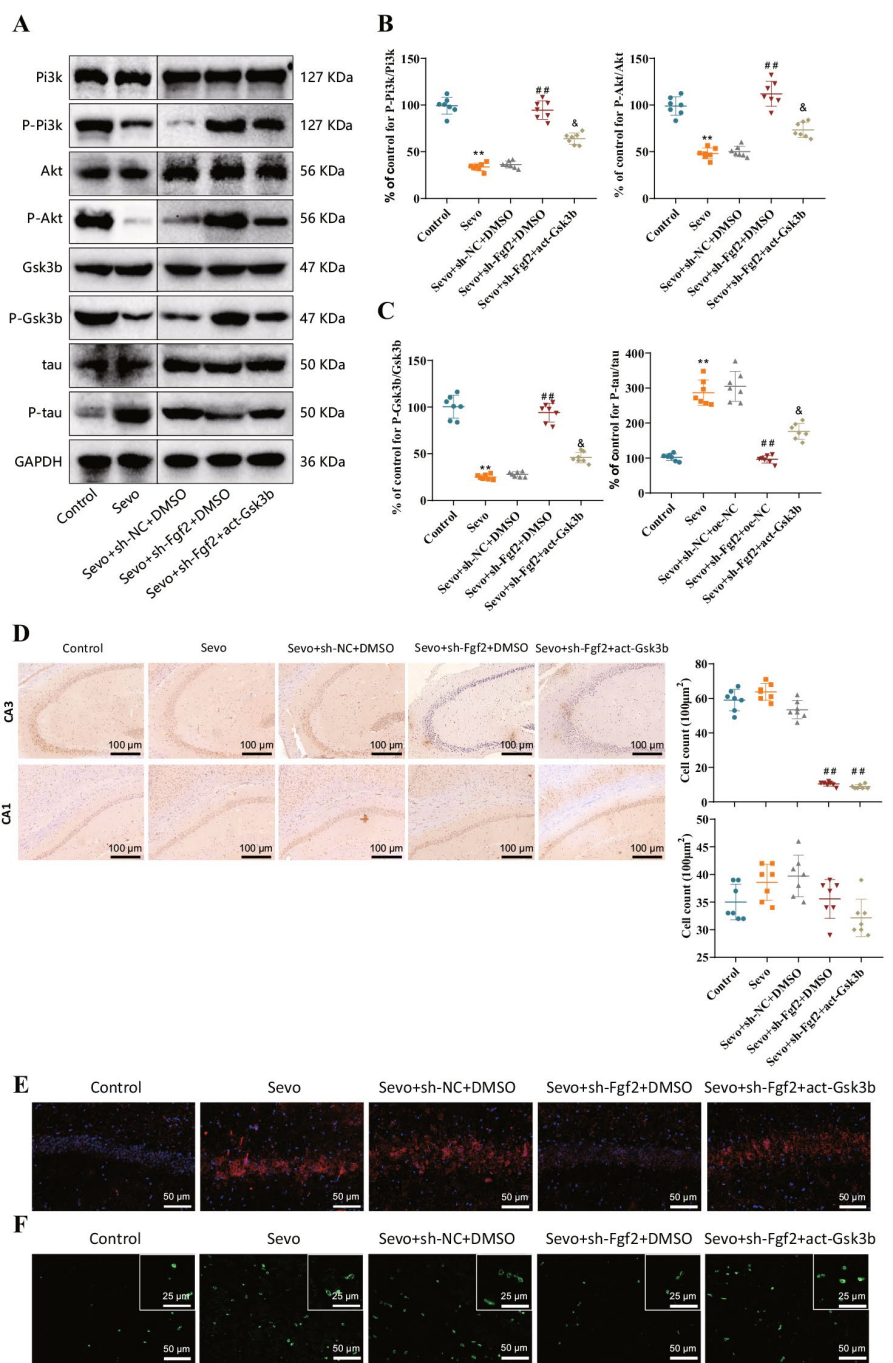
Fgf2 promotes abnormal accumulation of tau protein and neurofibrillary tangle formation by regulating Gsk3b. *Note*: (**A**) Western blot analysis of PI3K, P-PI3K, AKT, P-AKT, Gsk3b, P-Gsk3b, tau, and P-tau protein expression in Sevo model rats. (**B**) Statistical analysis of P-PI3K/PI3K and P-AKT/AKT protein expression ratios. (**C**) Statistical analysis of P-Gsk3b/Gsk3b and P-tau/tau protein expression ratios. (**D**) Immunohistochemistry and corresponding quantitative analysis of Fgf2 in the CA3 and CA1 regions confirmed the successful knockdown of Fgf2 in the CA3 region. (**E**) Immunofluorescence detection of P-tau content in hippocampal tissues of Sevo model rats. (**F**) Immunofluorescence detection of neurofibrillary tangles in hippocampal tissues of Sevo model rats. Sevo group compared to Control group, **P < 0.01; ^##^ indicates comparison between Sevo + sh-Fgf2 + DMSO or Sevo + sh-Fgf2 + act-Gsk3b group to the Sevo + sh-NC + DMSO groups, *P* < 0.01; & indicates comparison between Sevo + sh-Fgf2 + act-Gsk3b and Sevo + sh-Fgf2 + DMSO groups, *P* < 0.05. Each treatment group consisted of seven rats, and all experiments were repeated three times

Detection of phosphorylation levels of PI3K, AKT, Gsk3b, and tau in individual rats from different treatment groups by Western blot assay. The results showed that compared to the Control group, the phosphorylation levels of PI3K, AKT, and Gsk3b in the Sevo group rats decreased while the phosphorylation level of tau increased. Compared with the Sevo + sh-NC + DMSO group, the phosphorylation levels of PI3K, AKT, and Gsk3b in the Sevo + sh-Fgf2 + DMSO group of rats increased, while the phosphorylation level of tau decreased significantly. Compared to the Sevo + sh-Fgf2 + DMSO group, the phosphorylation levels of PI3K, AKT, and Gsk3b in rats from the Sevo + sh-Fgf2 + act-Gsk3b group were decreased, while the phosphorylation level of tau was increased (Fig. 6A–C).

After successfully knocking down Fgf2 in the CA3 region (Fig. 6D), further investigation was conducted to examine phosphorylation of tau protein and the occurrence of neurofibrillary tangles in hippocampal tissue using immunofluorescence experiments. The results demonstrated that compared to the Control group, rats in the Sevo group exhibited significantly increased levels of tau protein phosphorylation and exacerbated neurofibrillary tangles. In comparison to the Sevo + sh-NC + DMSO group, rats in the Sevo + sh-Fgf2 + DMSO group displayed a significant reduction in tau protein phosphorylation levels and a decrease in neurofibrillary tangles. However, rats in the Sevo + sh-Fgf2 + act-Gsk3b group exhibited a significant increase in tau protein phosphorylation levels and an exacerbation of neurofibrillary tangles compared to the Sevo + sh-Fgf2 + DMSO group (Fig. 6E, F).

The above results indicate that silencing Fgf2 could decrease the activity of Gsk3b and inhibit the phosphorylation of tau protein, thereby suppressing the formation of neurofibrillary tangles and improving sevoflurane-induced neurocognitive impairment.

## Discussion

The domain of biomedical research has witnessed remarkable progress, with high-throughput transcriptomics sequencing emerging as a potent instrument for unraveling the intricate molecular intricacies inherent to complex diseases (Seyed Tabib et al. 2020). In recent times, there has been a notable surge in scientific inquiries into age-related cognitive impairment, a debilitating condition significantly impacting the well-being of older adults (Feng et al. 2020; Porsteinsson et al. 2021; Wang et al. 2020b). This subject has gained considerable prominence within academic circles, with a particular focus on elucidating the possible linkages between anesthesia administration and the subsequent emergence of potential neurocognitive impairments (Lu et al. 2022). In this specific investigation, meticulous scrutiny was directed toward assessing the effects of sevoflurane, a widely employed anesthetic, on the cognitive capacities of rats. Employing advanced high-throughput transcriptomic sequencing methodologies, the study effectively unveiled the pivotal regulatory role played by the Fgf2 gene in this intricate process. This revelation not only furnishes fresh insights into the neurotoxic mechanisms associated with sevoflurane but also establishes a solid foundation for prospective therapeutic interventions.

Fgf2 has gained increasing recognition in recent years for its crucial role in influencing neural physiology and the disease processes that ensue (Vaseenon et al. 2020). In our ongoing study, a conspicuous increase in Fgf2 expression was noted within the aged rat model following exposure to sevoflurane (Wang et al. 2022). Remarkably, this surge in expression appears intricately tied to the activation of the PI3K/AKT/Gsk3b signaling pathway, a well-acknowledged participant in various cellular processes, encompassing growth, metabolism, and survival (Liu et al. 2021b). It is noteworthy that the heightened Fgf2 expression strongly correlates with elevated tau protein phosphorylation levels. Aberrations in tau protein phosphorylation constitute a distinctive hallmark evident in a multitude of neurodegenerative diseases, with Alzheimer’s disease taking a prominent stance among them (Cai et al. 2022). To further bolster the connection between Fgf2 and tau protein phosphorylation, we conducted a gene knockdown experiment with meticulous precision. Findings stemming from this experimental endeavor lend robust empirical support to the proposition that diminishing Fgf2 expression effectively hampers tau protein phosphorylation and ameliorates the formation of neurofibrillary tangles, pivotal pathological attributes associated with neurodegenerative disorders. Consequently, these findings provide compelling empirical evidence for Fgf2’s involvement in the intricate molecular mechanisms governing cognitive impairment, accentuating its potential as a pivotal target for future therapeutic interventions (Heuer et al. 2020).

The extensive landscape of neuroscience research has delved into the intricate relationship between Fgf2 and various neurological disorders, emphasizing its potential role in neural regeneration and repair processes. However, within the context of sevoflurane-induced cognitive impairment in the elderly, the precise contribution of Fgf2 has remained an enigma (Wang et al. 2021b). This inquiry is designed to bridge this critical gap in the scientific literature and unveil a definitive link connecting Fgf2 with tau protein phosphorylation. Our meticulous in vitro experiments leave no room for ambiguity, showcasing that sevoflurane exposure leads to a substantial increase in Fgf2 expression levels within rat cortical neurons. This surge in expression is orchestrated through the activation of the PI3K/AKT signaling pathway, which subsequently heightens the activity of the Gsk3b enzyme, ultimately resulting in the excessive phosphorylation of tau protein. Subsequent in vivo investigations affirm these findings by demonstrating that silencing Fgf2 effectively mitigates tau protein phosphorylation, curtails the formation of neurofibrillary tangles, and significantly ameliorates cognitive function in aged rats. These groundbreaking revelations furnish a novel framework for comprehending the molecular mechanisms that underlie sevoflurane-induced neurocognitive impairment, representing a noteworthy contribution to the existing corpus of scientific knowledge (Jiang 2022).

## Conclusion

**Fig. 7:**
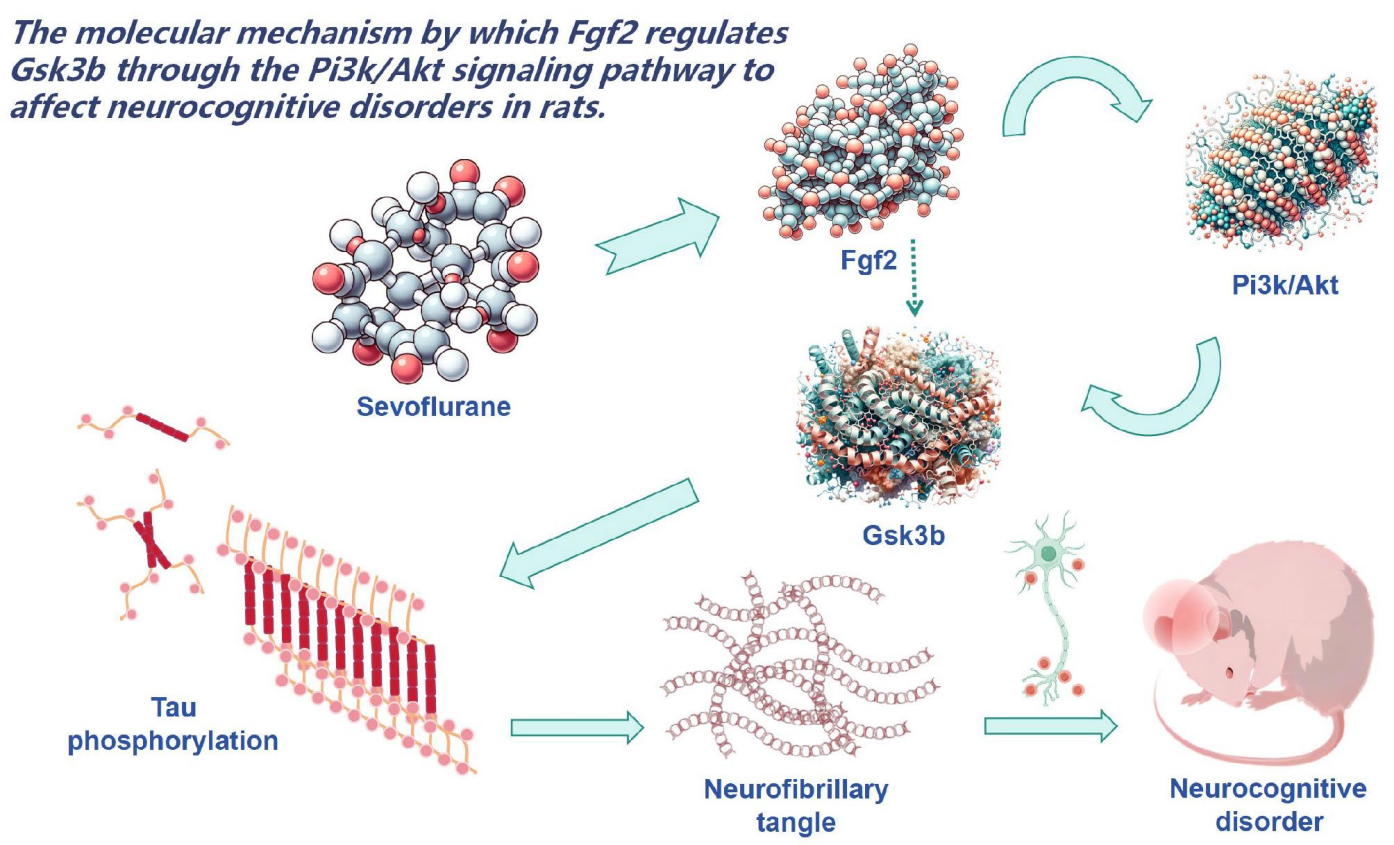
Molecular mechanisms underlying the effect of Fgf2 on Gsk3b and its impact on cognitive impairment in rats

Our research unfurls a compelling narrative wherein sevoflurane exposure exerts a profound influence on the heightened expression of Fgf2 within the physiology of aged rats (Fig. 7). Furthermore, our meticulous dissection of this phenomenon uncovers the intricate orchestration through the PI3K/AKT/Gsk3b signaling pathway, ultimately giving rise to an augmented phosphorylation of tau protein and the ensuing manifestation of cognitive impairment. This groundbreaking revelation not only ushers in a fresh perspective on the intricate interplay between anesthesia and age-related cognitive deficits but also illuminates the concealed molecular mechanisms underlying this relationship. Moreover, this pioneering discovery unfurls a hitherto unexplored therapeutic target within the clinical realm, promising advancements in future therapeutic approaches. However, it is incumbent upon us to recognize that our study grapples with certain inherent limitations. Specifically, the entirety of our experiments was confined to rodent models, engendering pertinent questions regarding the generalizability of these findings to the human populace. In the pursuit of a more comprehensive and finely-tuned understanding, forthcoming investigations should embark on the validation of this mechanism in human subjects and delve into supplementary therapeutic strategies intertwined with this newfound revelation.

### Electronic supplementary material

Below is the link to the electronic supplementary material.

Supplementary Material 1

Supplementary Material 2

Supplementary Material 3

## Data Availability

The datasets generated and/or analyzed during the current study are available from the corresponding author on reasonable request.

## Abbreviations

MWM: Morris Water Maze
GEO: Gene Expression Omnibus
DEGs: Differentially Expressed Genes
GO: Gene Ontology
PI3K: Phosphoinositide 3-kinase
AKT: Protein Kinase B
Gsk3b: Glycogen Synthase Kinase 3 beta
cDNA: Complementary DNA
BSA: Bovine Serum Albumin
PVDF: Polyvinylidene Difluoride
SDS-PAGE: Sodium Dodecyl Sulfate-Polyacrylamide Gel Electrophoresis
RT-qPCR: Reverse Transcription-quantitative Polymerase Chain Reaction
TBARS: Thiobarbituric Acid Reactive Substances
MDA: Malondialdehyde
GSH: Glutathione
ROS: Reactive Oxygen Species
DCFH-DA: 2,7-Dichlorodihydrofluorescein Diacetate
DHE: Dihydroethidium
ANOVA: Analysis of Variance

## References

[CR1] Amirghasemi F, Adjei-Sowah E, Pockaj BA, Nikkhah M (2021). Microengineered 3D Tumor models for Anti-cancer Drug Discovery in female-related cancers. Ann Biomed Eng.

[CR2] Andreollo NA, Santos EF, Araújo MR, Lopes LR (2012). Rat’s age versus human’s age: what is the relationship?. Arq Bras Cir Dig.

[CR3] Cai H, Luo Y, Yan X et al. The Mechanisms of Bushen-Yizhi Formula as a Therapeutic Agent against Alzheimer’s Disease. Sci Rep. 2018;8(1):3104. Published 2018 Feb 15. 10.1038/s41598-018-21468-w. 10.1038/s41598-018-21468-w PMC5814461 29449587

[CR5] Cai ZP, Cao C, Guo Z (2021). Coeloglossum viride var. Bracteatum extract attenuates staurosporine induced neurotoxicity by restoring the FGF2-PI3K/Akt signaling axis and Dnmt3. Heliyon.

[CR4] Cai H, Pang Y, Wang Q et al. Proteomic profiling of circulating plasma exosomes reveals novel biomarkers of Alzheimer’s disease. Alzheimers Res Ther. 2022;14(1):181. Published 2022 Dec 5. 10.1186/s13195-022-01133-1. 10.1186/s13195-022-01133-1 PMC9720984 36471423

[CR7] Chen XY, Wan SF, Yao NN et al. Inhibition of the immunoproteasome LMP2 ameliorates ischemia/hypoxia-induced blood-brain barrier injury through the Wnt/β-catenin signalling pathway. Mil Med Res. 2021a;8(1):62. Published 2021 Dec 3. 10.1186/s40779-021-00356-x. 10.1186/s40779-021-00356-x PMC8641178 34857032

[CR8] Chen Y, Li L, Zhang J (2021). Dexmedetomidine alleviates Lipopolysaccharide-Induced hippocampal neuronal apoptosis via inhibiting the p38 MAPK/c-Myc/CLIC4 signaling pathway in rats. Mol Neurobiol.

[CR6] Chen X, Wen J, Liu C, Guo D (2022). KLF4 downregulates FGF21 to activate inflammatory injury and oxidative stress of LPS–induced ATDC5 cells via SIRT1/NF–κB/p53 signaling. Mol Med Rep.

[CR9] Clemency BM, Varughese R, Gonzalez-Rojas Y (2022). Efficacy of inhaled ciclesonide for Outpatient treatment of adolescents and adults with symptomatic COVID-19: a Randomized Clinical Trial. JAMA Intern Med.

[CR10] Cooley SA, Nelson B, Boerwinkle A (2023). Plasma Aβ42/Aβ40 ratios in older people with Human Immunodeficiency Virus. Clin Infect Dis.

[CR11] Daniel D, Phillippi S, Schneider LJ, Nguyen KN, Mirpuri J, Lund AK (2021). Exposure to diesel exhaust particles results in altered lung microbial profiles, associated with increased reactive oxygen species/reactive nitrogen species and inflammation, in C57Bl/6 wildtype mice on a high-fat diet. Part Fibre Toxicol.

[CR12] Efferth T, Oesch F (2021). The immunosuppressive activity of artemisinin-type drugs towards inflammatory and autoimmune diseases. Med Res Rev.

[CR13] Enciso J, Mendoza L, Álvarez-Buylla ER, Pelayo R (2020). Dynamical modeling predicts an inflammation-inducible CXCR7 + B cell precursor with potential implications in lymphoid blockage pathologies. PeerJ.

[CR14] Fang M, Yin Y, Chen H, Hu Z, Davies H, Ling S (2013). Contribution of Rag1 to spatial memory ability in rats. Behav Brain Res.

[CR15] Feng YS, Yang SD, Tan ZX (2020). The benefits and mechanisms of exercise training for Parkinson’s disease. Life Sci.

[CR16] Geschwind DH (2003). Tau phosphorylation, tangles, and neurodegeneration: the chicken or the egg?. Neuron.

[CR17] Gómez-Ramos A, Smith MA, Perry G, Avila J (2004). Tau phosphorylation and assembly. Acta Neurobiol Exp (Wars).

[CR18] Harmer B, Lee S, Duong TVH, Saadabadi A. Suicidal ideation. StatPearls. Treasure Island (FL). Volume 24. StatPearls Publishing; 2023.

[CR19] He Q, Li Z, Meng C, Wu J, Zhao Y, Zhao J (2019). Parkin-dependent Mitophagy is required for the inhibition of ATF4 on NLRP3 inflammasome activation in Cerebral Ischemia-Reperfusion Injury in rats. Cells.

[CR20] Hernandez F, Lucas JJ, Avila J (2013). GSK3 and tau: two convergence points in Alzheimer’s disease. J Alzheimers Dis.

[CR21] Heuer SE, Neuner SM, Hadad N (2020). Identifying the molecular systems that influence cognitive resilience to Alzheimer’s disease in genetically diverse mice. Learn Mem.

[CR22] Hong XP, Chen T, Yin NN (2016). Puerarin ameliorates D-Galactose Induced enhanced hippocampal neurogenesis and tau hyperphosphorylation in rat brain. J Alzheimers Dis.

[CR23] Jiang Y (2022). Osteoarthritis year in review 2021: biology. Osteoarthritis Cartilage.

[CR24] Josephs KA, Whitwell JL, Tosakulwong N (2015). TAR DNA-binding protein 43 and pathological subtype of Alzheimer’s disease impact clinical features. Ann Neurol.

[CR25] Karamanavi E, McVey DG, van der Laan SW (2022). The FES Gene at the 15q26 coronary-artery-disease locus inhibits atherosclerosis. Circ Res.

[CR26] Kiyota T, Ingraham KL, Jacobsen MT, Xiong H, Ikezu T. FGF2 gene transfer restores hippocampal functions in mouse models of Alzheimer’s disease and has therapeutic implications for neurocognitive disorders [published correction appears in Proc Natl Acad Sci U S A. 2011;108(52):21282]. Proc Natl Acad Sci U S A. 2011;108(49):E1339-E1348. 10.1073/pnas.1102349108. 10.1073/pnas.1102349108 PMC3241747 22042871

[CR27] Kotekar N, Shenkar A, Nagaraj R (2018). Postoperative cognitive dysfunction - current preventive strategies. Clin Interv Aging.

[CR28] Krüger K (2018). Medikamentöse Therapie Der Rheumatoiden Arthritis und ihrer Komorbiditäten [Pharmacological treatment of rheumatoid arthritis and its comorbidities]. Internist (Berl).

[CR29] Lai Y, Lin P, Chen M (2020). Restoration of L-OPA1 alleviates acute ischemic stroke injury in rats via inhibiting neuronal apoptosis and preserving mitochondrial function. Redox Biol.

[CR30] Lamberty Y, Gower AJ (1991). Simplifying environmental cues in a Morris-type water maze improves place learning in old NMRI mice. Behav Neural Biol.

[CR31] Li C, Zhao Z, Luo Y (2021). Macrophage-disguised Manganese Dioxide nanoparticles for Neuroprotection by reducing oxidative stress and modulating Inflammatory Microenvironment in Acute ischemic stroke. Adv Sci (Weinh).

[CR32] Li X, Zhang H, Yang L (2023). Inhibition of NLRP1 inflammasome improves autophagy dysfunction and Aβ disposition in APP/PS1 mice. Behav Brain Funct.

[CR33] Liao J, Chen G, Liu X (2022). C/EBPβ/AEP signaling couples atherosclerosis to the pathogenesis of Alzheimer’s disease. Mol Psychiatry.

[CR34] Lin SY, Wang YY, Chang CY et al. Effects of β-Adrenergic Blockade on Metabolic and Inflammatory Responses in a Rat Model of Ischemic Stroke. Cells. 2020a;9(6):1373. Published 2020 Jun 1. 10.3390/cells9061373. 10.3390/cells9061373 PMC7349353 32492962

[CR36] Lin X, Chen Y, Zhang P, Chen G, Zhou Y, Yu X (2020). The potential mechanism of postoperative cognitive dysfunction in older people. Exp Gerontol.

[CR35] Lin SY, Wang YY, Chang CY (2021). TNF-α receptor inhibitor alleviates metabolic and inflammatory changes in a rat model of ischemic stroke. Antioxid (Basel).

[CR37] Liu Q, Dong H, Zhao W (2021). Design, synthesis, and Biological evaluation of APN and AKT Dual-Target inhibitors. ACS Med Chem Lett.

[CR38] Liu W, Qaed E, Zhu HG, Dong MX, Tang Z (2021). Non-energy mechanism of phosphocreatine on the protection of cell survival. Biomed Pharmacother.

[CR39] Lu J, Hou W, Gao S, Zhang Y, Zong Y (2022). The role of Gut Microbiota-Gut-Brain Axis in Perioperative Neurocognitive Dysfunction. Front Pharmacol.

[CR40] McCrea MA, Giacino JT, Barber J (2021). Functional outcomes over the First Year after moderate to severe traumatic brain Injury in the prospective, longitudinal TRACK-TBI study. JAMA Neurol.

[CR41] Müller AR, Zinkstok JR, Rommelse NNJ et al. Methylphenidate for attention-deficit/hyperactivity disorder in patients with Smith-Magenis syndrome: protocol for a series of N-of-1 trials. Orphanet J Rare Dis. 2021;16(1):380. Published 2021 Sep 8. 10.1186/s13023-021-02003-z. 10.1186/s13023-021-02003-z PMC8424817 34496899

[CR42] Narayan AJ, Lieberman AF, Masten AS (2021). Intergenerational transmission and prevention of adverse childhood experiences (ACEs). Clin Psychol Rev.

[CR43] Ni C, Li Z, Qian M, Zhou Y, Wang J, Guo X (2015). Isoflurane induced cognitive impairment in aged rats through hippocampal calcineurin/NFAT signaling. Biochem Biophys Res Commun.

[CR44] Ojeda L, Gao J, Hooten KG (2011). Critical role of PI3K/Akt/GSK3β in motoneuron specification from human neural stem cells in response to FGF2 and EGF. PLoS ONE.

[CR45] Pant T, DiStefano JK, Logan S, Bosnjak ZJ (2021). Emerging role of long noncoding RNAs in Perioperative Neurocognitive disorders and Anesthetic-Induced Developmental Neurotoxicity. Anesth Analg.

[CR46] Porsteinsson AP, Isaacson RS, Knox S, Sabbagh MN, Rubino I (2021). Diagnosis of early Alzheimer’s Disease: clinical practice in 2021. J Prev Alzheimers Dis.

[CR47] Ritchie ME, Phipson B, Wu D (2015). Limma powers differential expression analyses for RNA-sequencing and microarray studies. Nucleic Acids Res.

[CR48] Rogawski DS, Vitanza NA, Gauthier AC, Ramaswamy V, Koschmann C (2017). Integrating RNA sequencing into neuro-oncology practice. Transl Res.

[CR49] Saranteas T, Spiliotaki H, Koliantzaki I (2019). The utility of Echocardiography for the prediction of spinal-Induced Hypotension in Elderly patients: Inferior Vena Cava Assessment is a key player. J Cardiothorac Vasc Anesth.

[CR50] Scheltens P, De Strooper B, Kivipelto M (2021). Alzheimer’s disease. Lancet.

[CR51] Ellinghaus D, Degenhardt F, Severe Covid-19 GWAS Group (2020). Genomewide Association Study of Severe Covid-19 with respiratory failure. N Engl J Med.

[CR52] Seyed Tabib NS, Madgwick M, Sudhakar P, Verstockt B, Korcsmaros T, Vermeire S (2020). Big data in IBD: big progress for clinical practice. Gut.

[CR53] Shen M, Lian N, Song C, Qin C, Yu Y, Yu Y (2021). Different anesthetic drugs mediate changes in Neuroplasticity during Cognitive Impairment in Sleep-deprived rats via different factors. Med Sci Monit.

[CR54] Smadja DM, Mentzer SJ, Fontenay M (2021). COVID-19 is a systemic vascular hemopathy: insight for mechanistic and clinical aspects. Angiogenesis.

[CR55] Tanaka T, Tsujio I, Nishikawa T, Shinosaki K, Kudo T, Takeda M (2000). Significance of tau phosphorylation and protein kinase regulation in the pathogenesis of Alzheimer disease. Alzheimer Dis Assoc Disord.

[CR56] Tatebayashi Y, Iqbal K, Grundke-Iqbal I (1999). Dynamic regulation of expression and phosphorylation of tau by fibroblast growth factor-2 in neural progenitor cells from adult rat hippocampus. J Neurosci.

[CR58] Tian Y, Chen KY, Liu LD, Dong YX, Zhao P, Guo SB. Sevoflurane Exacerbates Cognitive Impairment Induced by Aβ^1–40^ in Rats through Initiating Neurotoxicity, Neuroinflammation, and Neuronal Apoptosis in Rat Hippocampus. Mediators Inflamm. 2018;2018:3802324. Published 2018 Oct 9. 10.1155/2018/3802324. 10.1155/2018/3802324 PMC6198580 30402039

[CR57] Tian H, Ding N, Guo M et al. Analysis of Learning and Memory Ability in an Alzheimer’s Disease Mouse Model using the Morris Water Maze. J Vis Exp. 2019;(152):10.3791/60055. Published 2019 Oct 29. 10.3791/60055. 10.3791/60055 31736488

[CR59] Tian Y, Liu B, Li Y (2022). Activation of RARα receptor attenuates Neuroinflammation after SAH via promoting M1-to-M2 phenotypic polarization of Microglia and regulating Mafb/Msr1/PI3K-Akt/NF-κB pathway. Front Immunol.

[CR60] Van Meter AR, Perez-Rodriguez MM, Braga RJ (2021). Pramipexole to improve cognition in bipolar disorder: a Randomized Controlled Trial. J Clin Psychopharmacol.

[CR61] Vaseenon S, Chattipakorn N, Chattipakorn SC (2020). The possible role of basic fibroblast growth factor in dental pulp. Arch Oral Biol.

[CR65] Wang Y, Qian M, Qu Y (2020). Genome-wide screen of the Hippocampus in aged rats identifies Mitochondria, metabolism and aging processes implicated in Sevoflurane Anesthesia. Front Aging Neurosci.

[CR63] Wang F, Kream RM, Stefano GB (2020). Long-term respiratory and neurological sequelae of COVID-19. Med Sci Monit.

[CR64] Wang P, Yin X, Chen G (2021). Perioperative probiotic treatment decreased the incidence of postoperative cognitive impairment in elderly patients following non-cardiac surgery: a randomised double-blind and placebo-controlled trial. Clin Nutr.

[CR62] Wang CM, Chen WC, Zhang Y, Lin S, He HF (2021). Update on the mechanism and treatment of Sevoflurane-Induced Postoperative Cognitive Dysfunction. Front Aging Neurosci.

[CR66] Wang Z, Wang Z, Wang A (2022). The neuroprotective mechanism of sevoflurane in rats with traumatic brain injury via FGF2. J Neuroinflammation.

[CR67] Wegmann S, Biernat J, Mandelkow E (2021). A current view on tau protein phosphorylation in Alzheimer’s disease. Curr Opin Neurobiol.

[CR70] Xu Z, Dong Y, Wang H (2014). Age-dependent postoperative cognitive impairment and Alzheimer-related neuropathology in mice. Sci Rep.

[CR69] Xu L, Shen J, Yu L (2018). Role of autophagy in sevoflurane-induced neurotoxicity in neonatal rat hippocampal cells. Brain Res Bull.

[CR68] Xu B, Xu J, Cai N (2021). Roflumilast prevents ischemic stroke-induced neuronal damage by restricting GSK3β-mediated oxidative stress and IRE1α/TRAF2/JNK pathway. Free Radic Biol Med.

[CR71] Yang Y, Wang L, Zhang C (2022). Ginsenoside Rg1 improves Alzheimer’s disease by regulating oxidative stress, apoptosis, and neuroinflammation through Wnt/GSK-3β/β-catenin signaling pathway. Chem Biol Drug Des.

[CR73] Zhang F, Peng WX, Wang L (2018). Role of FGF-2 transfected bone marrow mesenchymal stem cells in Engineered Bone tissue for repair of avascular necrosis of femoral head in rabbits. Cell Physiol Biochem.

[CR74] Zhang JH, Yu LJ, Yang H et al. Huatuo Zaizao pill ameliorates cognitive impairment of APP/PS1 transgenic mice by improving synaptic plasticity and reducing Aβ deposition. BMC Complement Altern Med. 2018b;18(1):167. Published 2018 May 29. 10.1186/s12906-018-2237-2. 10.1186/s12906-018-2237-2 PMC5975403 29843688

[CR72] Zhang C, Feng YG, Tam C, Wang N, Feng Y (2021). Transcriptional profiling and machine learning unveil a concordant biosignature of type I Interferon-Inducible Host Response across Nasal Swab and pulmonary tissue for COVID-19 diagnosis. Front Immunol.

[CR75] Zhou Z, Hou J, Mo Y (2020). Geniposidic acid ameliorates spatial learning and memory deficits and alleviates neuroinflammation via inhibiting HMGB-1 and downregulating TLR4/2 signaling pathway in APP/PS1 mice. Eur J Pharmacol.

[CR76] Zhu X, Qiu C, Wang Y (2022). FGFR1 SUMOylation coordinates endothelial angiogenic signaling in angiogenesis. Proc Natl Acad Sci U S A.

