# Supplementary material for: Involvement of Fgf2-mediated tau protein phosphorylation in cognitive deficits induced by sevoflurane in aged rats
Source: Mol Med. 2024 Mar 16;30:39. doi: 10.1186/s10020-024-00784-0 (PMC10943822; doi:10.1186/s10020-024-00784-0)
Supplement: Supplementary file 1 — Supplementary Material 1 [file 10020_2024_784_MOESM1_ESM.docx]

**Table S1. shRNA interference sequence**

| Name | shRNA Sequences (5’-3’) |
| --- | --- |
| sh-NC | CCTAAGGTTAAGTCGCCCTCG |
| sh-Fgf2-1 | GCTTCTAAGTGTGTTACAGAA |
| sh-Fgf2-2 | CACGTCAAACTACAACTCCAA |

**Table S2. The RT-qPCR primer sequence**

| Genes | Sequences (5’-3’) |
| --- | --- |
| Fgf2 (rat) | F: CAAAACCTGACCCGATCCCT |
|  | R: CCGTGACGCAGCTCCTAAA |
| TNF-α (rat) | F: GGCGTGTTCATCCGTTCTCT |
|  | R: CCCAGAGCCACAATTCCCTT |
| IL-6 (rat) | F: TTCCAGCCAGTTGCCTTCTT |
|  | R: TGAAGTCTCCTCTCCGGACT |
| IL-1β (rat) | F: CCTATGTCTTGCCCGTGGAG |
|  | R: CACACACTAGCAGGTCGTCA |
| Gapdh (rat) | F: AGACAGCCGCATCTTCTTGT |
|  | R: TACGGCCAAATCCGTTCACA |

Note: F, forward; R, reverse.

**Table S3. Western blot antibody information**

| Target name | Manufacturer | Product code | Dilution ratio |
| --- | --- | --- | --- |
| Fgf2 (rat) | Thermo Fisher | PA5-116495 | 1:500 |
| Pi3k (rat) | Abcam | ab302958 | 1:1000 |
| Akt (rat) | Abcam | ab8805 | 1:500 |
| Gsk3b (rat) | Abcam | ab93926 | 1:1000 |
| P-tau (rat) | Thermo Fisher | 701530 | 1:5000 |
| tau (rat) | Abcam | ab76128 | 1:10000 |
| Gapdh (rat) | Abcam | ab181602 | 1:10000 |
| P-Pi3k (rat) | Abcam | ab182651 | 1:500 |
| P-Akt (rat) | Abcam | ab38449 | 1:1000 |
| P-Gsk3b (rat) | Thermo Fisher | MA5-14873 | 1:1000 |
